# The impact of eHealth use on general practice workload in the pre-COVID-19 era: a systematic review

**DOI:** 10.1186/s12913-024-11524-9

**Published:** 2024-09-19

**Authors:** Jelle Keuper, Lilian H. D. van Tuyl, Ellemarijn de Geit, Corinne Rijpkema, Elize Vis, Ronald Batenburg, Robert Verheij

**Affiliations:** 1 Netherlands Institute for Health Services Research (NIVEL), Otterstraat 118, Utrecht, 3513CR Netherlands; 2 Tranzo, Tilburg School of Social and Behavioral Sciences, Tilburg University, Professor Cobbenhagenlaan 125, Tilburg, 5037DB Netherlands; 3 Department of Sociology, Radboud University Nijmegen, Thomas van Aquinostraat 4, Nijmegen, 6525GD Netherlands

**Keywords:** eHealth, General practice, Workload, Impact, Systematic review

## Abstract

**Background:**

In recent years, eHealth has received much attention as an opportunity to increase efficiency within healthcare organizations. Adoption of eHealth might consequently help to solve perceived health workforce challenges, including labor shortages and increasing workloads among primary care professionals, who serve as the first point of contact for healthcare in many countries.

The purpose of this systematic review was to investigate the impact of general eHealth use and specific eHealth services use on general practice workload in the pre-COVID-19 era.

**Methods:**

The databases of CINAHL, Cochrane, Embase, IEEE Xplore, Medline ALL, PsycINFO, Web of Science, and Google Scholar were searched, using combinations of keywords including ‘eHealth’, ‘workload’, and ‘general practice’. Data extraction and quality assessment of the included studies were independently performed by at least two reviewers. Publications were included for the period 2010 – 2020, before the start of the COVID-19 pandemic.

**Results:**

In total, 208 studies describing the impact of eHealth services use on general practice workload were identified. We found that two eHealth services were mainly investigated within this context, namely electronic health records and digital communication services, and that the largest share of the included studies used a qualitative study design. Overall, a small majority of the studies found that eHealth led to an increase in general practice workload. However, results differed between the various types of eHealth services, as a large share of the studies also reported a reduction or no change in workload.

**Conclusions:**

The impact of eHealth services use on general practice workload is ambiguous. While a small majority of the effects indicated that eHealth increased workload in general practice, a large share of the effects also showed that eHealth use reduced workload or had no impact. These results do not imply a definitive conclusion, which underscores the need for further explanatory research. Various factors, including the study setting, system design, and the phase of implementation, may influence this impact and should be taken into account when general practices adopt new eHealth services.

**Study registration number:**

PROSPERO (International Prospective Register of Systematic Reviews) CRD42020199897; https://www.crd.york.ac.uk/prospero/display_record.php?RecordID=199897.

**Supplementary Information:**

The online version contains supplementary material available at 10.1186/s12913-024-11524-9.

## Background

Within healthcare, the adoption of eHealth has received much attention in recent years as it promises, amongst others, to increase efficiency, improve quality, enable patient empowerment, encourage the patient and professional relationship, and make healthcare more equitable accessible, and of sufficient quality [1]. The term eHealth was barely used before 1999 and is defined by Eysenbach et al. in 2001 as *“an emerging field in the intersection of medical informatics, public health and business, referring to health services and information delivered or enhanced through the Internet and related technologies. In a broader sense, the term characterizes not only a technical development, but also a state-of-mind, a way of thinking, an attitude, and a commitment for networked, global thinking, to improve health care locally, regionally, and worldwide by using information and communication technology”* [1]. During the last two decades, this definition has been regularly used and cited in scientific literature. Since then, several other definitions of eHealth have emerged, various eHealth categorizations or taxonomies have been developed, and several types of eHealth services have been introduced and increasingly used in the various healthcare sectors that exist worldwide, including general practice care [2–9].

During the last decades, global healthcare has been confronted with several challenges, including aging populations, increasing prevalence of multimorbidity, the substitution of care, and recurring pandemics, causing healthcare staffing shortages [10–12]. These shortages are expected to rise even further in the coming years, causing increased workloads among healthcare professionals, such as general practice or primary care practice staff, which is usually the first-point-of-contact for the general population [13–15].

The potential increase in efficiency as a result of eHealth implementation and use within healthcare might help to solve these challenges. For example, Kochendorfer et al. [16] showed that shortly after the implementation of an eHealth application (i.e., an electronic health record (EHR)-generated rounding report), residents and attending physicians reported daily time savings of 44 min, indicating increased efficiency and a reduction in workload [16]. More recently, Ruiz Morilla et al. [17] also found that primary care physicians considered that eHealth (i.e., telemedicine) use would also improve their professional workload [17]. On the other hand, Adler et al. (2015) found in their study that the majority of family physicians who switched to a new EHR disagreed that it improved their productivity [18]. Further, Farr et al. (2018) showed that the use of an eHealth service (i.e., e-consultations) resulted in a duplication of general practitioners’ (GPs) workload. Besides these findings, other review studies investigating the impact of specific eHealth services (i.e., electronic clinical decision support tools and video consultation) within general practice care have reported mixed results regarding their influence on workload [19, 20].

During the last two decades, various research has been performed to study the impact of eHealth services use on healthcare and general practice staff workload. These studies particularly address the impact of one specific eHealth service on workload. Moreover, the studies performed during the COVID-19 pandemic are complicated by the pandemic’s impact on healthcare organization and the need to implement eHealth services in a fast and enforced manner. This makes reliable comparisons between findings on the impact of eHealth on healthcare professional workload from the pre-COVID-19 era and those from the COVID-19 period nearly impossible. A current omission in this field of research is a systematic analysis of the impact *across* the use of various types of eHealth services. This systematic review paper consequently aims to provide an overview of studies on the impact of eHealth services use on workload in general practices in the pre-COVID-19 era. This overview may also provide relevant insights for the future (i.e., post-COVID-19 era) implementation of eHealth services in general practice. We not only provide an overview of what can be concluded about the impact of both general and specific eHealth services use on general practice workload but also conduct a comparative analysis of these studies published in the period 2010 and April 2020, i.e. before the start of the COVID-19 pandemic.

## Methods

### Study Registration

The PRISMA-P (Preferred Reporting Items for Systematic Reviews and Meta-Analyses Protocols) statement, including its checklist, was used as guidance for performing this systematic review [21]. The review methods used were determined in advance, and the study was registered at PROSPERO (International Prospective Register of Systematic Reviews; CRD42020199897) to promote transparency about how this review was performed. The final data collection and analysis process did not require major adjustments compared to the initially determined methodology. For example, we decided at a later stage of our research process that we would use the Mixed Methods Appraisal Tool (MMAT) for the quality assessment procedure, as we think that this tool matched well with the results of our literature search. Furthermore, two reviewers (EdG and EH) were included at a later stage of the study (i.e., at the data extraction, data analysis and quality assessment process). Finally, we decided to extract additional data from included references, which we thought were relevant.

### Search strategy

The search string was composed with the help of a medical information specialist. The electronic databases of CINAHL, Cochrane, Embase, IEEE Xplore, Medline ALL, PsycINFO, Web of Science, and Google Scholar, as grey literature source, were systematically searched for relevant papers published in English in April 2020. Only original, empirical, peer-reviewed studies investigating the impact of eHealth services implementation and use on general practice workload, which were published between January 2010 and April 2020, were included in this systematic review. Other types of studies or papers, including special reports, conference abstracts, letters, commentaries, protocols, animal studies or clinical studies involving humans (e.g., randomized clinical trials) were excluded. Review articles were not excluded in this initial search.

The endpoint of this timeframe (e.g., April 2020) was deliberately chosen as the COVID-19 pandemic has radically changed the organization of healthcare and the perceived workload in general practices during a short and disruptive period. Through this selection, we avoid making comparisons between pre-COVID-19 pandemic era results and findings from studies that were performed during the pandemic or post-pandemic. The restrictive measures and their disruptive influence on our specific subject of study would make reliable comparisons between findings from studies conducted during these different periods complicated. Nonetheless, we believe that the results of our study may offer valuable insights for the future adoption of eHealth services in general practice. This is particularly relevant regarding the impact of eHealth on healthcare professionals, now that governmental restrictive measures have largely ended in most countries and no longer significantly affect healthcare organizations. Further, 2010 was chosen as the starting point of our search, as technology has rapidly changed, making results from before 2010 potentially outdated. Accordingly, articles including COVID-related terms, i.e. ‘covid’ or ‘corona’, were excluded. The electronic databases were searched for original papers, within the specified timeframe, containing terms related to all of the following main terms: ‘eHealth’, ‘workload’, and ‘general practice’. Therefore, related Medical Subject Headings (MeSH) terms were used as well as additional relevant non-MeSH terms, to select the full range of potentially appropriate original papers.

The search string and search strategy results are shown in Additional file 1, which were developed according to the participants, intervention, comparators, and outcomes (PICO) framework. With regard to the participants (P), we concentrated on studies describing staff working in general practice care or in a related setting (e.g., family medicine care or (community-based) primary healthcare). For convenience reasons, we will use the term ‘general practice’ in this paper, which for example also includes the term ‘family medicine’ which is more often used in North- and South American countries. Studies including participants from multiple care settings involving general practice staff were also included in our systematic review. With regard to the intervention (I), we included studies that described the implementation or use of one or more eHealth services within general practice care. The use of text messages, such as SMS, and telephone consultations were not considered eHealth in this study. Finally, the primary study outcome in our systematic review is ‘workload’. Secondary study outcome measures, related to workload, were also included and can be found in the search string included in Additional file 1.

### Study selection process

The reference management software EndNote X9 was used to import articles identified through our search strategy and to manage references for performing deduplication, abstract and title screening, and full-article screening, following the procedure described by Bramer et al. (2017) [22]. As a first stage of the abstract and title selection process, the first 100 references were sorted on the first author’s name. These articles were independently assessed by two reviewers (JK and EV) to determine a good strategy for selecting the right studies for the full-article screening process. Consequently, the results of this assessment were discussed afterwards and led to tightening of the abstract and title selection criteria. Afterwards, one reviewer (JK) independently screened all references on title and abstract and the other reviewer (EV) independently screened 20% of all references. After comparing the results of this abstract and title screening process, the interrater reliability was 98% and the kappa was 0.84, which can be considered as ‘near perfect’ according to Landis et al. (1977), which justifies the approach that only one reviewer screened all references during title and abstract screening, while the second reviewer screened a 20% sample [23]. Furthermore, in case of doubt whether or not to include a specific article for the full-text selection process, this article was discussed between the two reviewers.

The full-text selection process was also independently performed by two reviewers (JK and CR). One reviewer (JK) again screened all articles that remained after the abstract and title selection process, while the other reviewer (CR) screened a 20% sample of these records. Identified review articles were searched for further eligible studies, using the snowball method, and were finally excluded as these did not contain original data. This resulted in an interrater reliability of 89% and a kappa value of 0.90, which can also be considered as ‘near perfect’. In both selection processes, other reviewers (RB, LvT, RV) were consulted when disagreement or indistinctness persisted.

**Fig. 1:**
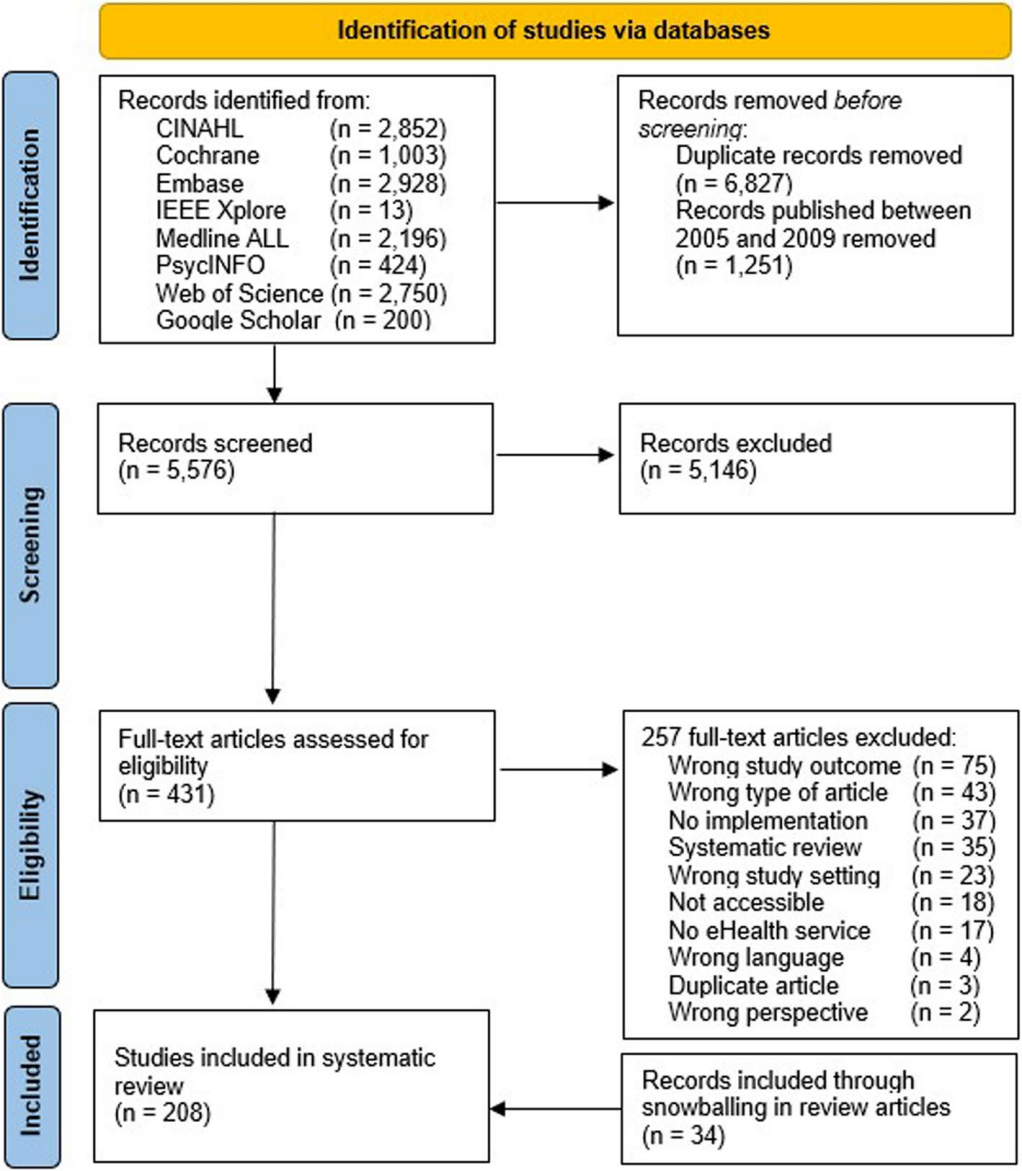
PRISMA flow diagram

Finally, through this process, references were included if they described at least one eHealth service, that was implemented and/or used within a general practice care setting, and investigated its impact on the workload within this setting. Reasons for exclusion were recorded in Endnote and are presented in the PRISMA flow diagram (Fig. 1).

### Data extraction

References that met all of the three aforementioned criteria were imported into a structured Microsoft Excel spreadsheet, which contained the following primary study characteristics and additional extracted details of the included references: author, year of publication, study design, study objective, outcome measures, primary and secondary findings, type of eHealth service, study population characteristics, and country of study. The PRISMA-P 2015 Explanation and Elaboration paper by Shamseer et al. [24] was used to guide the process of data extraction [24]. Data extraction was performed independently by three reviewers (JK, EH and EdG). Each reviewer again selected a 20% sample of the extracted articles, which were reassessed by another reviewer and discussed to reduce errors in data extraction and to determine which data should be extracted. If disagreement or indistinctness persisted a fourth reviewer (LvT) was consulted.

### Data analysis and quality assessment

A meta-analysis was not performed as the outcome measures and study designs varied widely among the included studies. Consequently, we conducted a descriptive analysis to assess the overall impact of eHealth use on workload in general practice, and to investigate the impact of specific eHealth services use on general practice workload. Therefore, extracted outcome measures and primary and secondary findings were assessed by the reviewers (JK, EH and EdG). If a study showed that the eHealth service increased general practice workload, a ‘ + ’ effect indicator was assigned to this study. A ‘- ‘ was given to studies reporting general practice workload reductions because of eHealth use. If studies reported that there was no impact of eHealth service use on workload, a ‘0’ was assigned. In case more than one effect was found in one study (e.g., a ‘ + ’ and ‘- ‘, or a ‘0’, ‘ + ’ and ‘- ‘), this study was assigned two or a maximum of three effect indicators. Further, no distinction was made based on the strength of the effect, as included studies had mainly slightly different study designs, settings and outcomes, which made comparisons of the effects between studies difficult.

In addition, quality assessment was independently performed by three reviewers (JK, EH and EdG) using the MMAT 2018, to appraise the methodological quality of the included studies [25]. For 20% of the included studies, a second reviewer also independently appraised the quality of these studies, with disagreements resolved through discussion until agreement was reached.

## Results

### Study Selection

The initial search identified 12,366 potentially relevant published articles, of which 431 articles were selected for the full-text screening (see Fig. 1). Finally, 208 articles remained after the full-text screening, describing 16 different categories of eHealth services within general practice settings (see Table 1). Details of the conducted searches can be found in Additional file 1.

**Table 1:** Studies by type of eHealth service and their assigned effects on general practice workload

Type of eHealth service	Number of times studied^*^	Number of effects assigned	+ ^a^ n (%)	-^b^ n (%)	0^c^ n (%)
EHR	68	87	54 (62)	23 (26)	10 (11)
Digital communication	40	58	27 (47)	17 (29)	14 (24)
Digital decision support	19	25	13 (52)	9 (36)	3 (12)
Telemonitoring	16	17	13 (76)	3 (18)	1 (6)
Mobile health (mHealth)	13	19	13 (68)	5 (26)	1 (5)
Electronic prescribing	13	18	8 (44)	10 (56)	0 (0)
General health information technology	9	11	4 (36)	5 (45)	2 (18)
Patient portal/patient online access	9	15	5 (33)	6 (4)	4 (27)
Health information exchange (HIE)	6	10	5 (50)	5 (50)	0 (0)
Algorithms or Artificial Intelligence (AI)	5	9	3 (33)	4 (44)	2 (22)
Digital mental healthcare	5	6	3 (50)	1 (17)	2 (33)
Digital patient questionnaire (screening) tool	4	6	1 (17)	2 (33)	3 (50)
Online test ordering	3	4	2 (50)	2 (50)	0 (0)
Teledermatology	3	4	2 (50)	2 (50)	0 (0)
Self-management tools	2	2	2 (100)	0 (0)	0 (0)
Other eHealth services	11	13	5 (38)	5 (38)	3 (23)
**Total**	**226**^**d**^	**304**	**162 (53)**	**99 (33)**	**44 (15)**

^*^The number of studied eHealth services is lower than the number of effects assigned, because some studies reported more than one effect of eHealth service use on general practice workload (see the data analysis section)

^a^ Number of references reporting an increase in workload as a result of eHealth service use

^b^ Number of references reporting a reduction in workload as a result of eHealth service use

^c^ Number of references reporting no effect on workload as a result of eHealth service use

^d^ Because some references studied more than one eHealth service type, this number is higher than the 208 included references

**Table:** 

**Specification of the eHealth service categories: ** The **EHR** category includes EHRs, health management information systems, electronic medical records (EMRs), and EHR/EMR functionalities, tasks or alerts. The **digital communication** category includes e-visits, (secure) emails, e-consultations, video consultations, video conferencing, webinars, electronic communication, online consultation systems, secure messaging, virtual communication, teleconsultations, e-appointments, e-referrals, and online request renewal of prescription medications. The **digital decision support** category includes computerized (clinical) decision support/aid tools, clinical pathways applications, computer-supported telephone triage, digital screening and surveillance applications, and electronic optional guideline tools. The **telemonitoring** category includes remote monitoring services and internet-mediated health and lifestyle programs. The **mHealth** category includes mobile health applications, mobile health components, mobile devices, mobile health systems, and mobile client data applications. The **electronic prescribing** category includes electronic prescribing tools or functionalities. The **general health information technology** category includes home(-based) telehealth, clinical pathways websites, Health Information Technology (HIT), and web-based counseling tools. The **patient portal/patient online access** category includes patient online (record) access, electronic patient portals, digital health information systems for citizens, and personal health records. The **Health Information Exchange (HIE)** category includes HIE systems and functionalities. The **algorithms or Artificial Intelligence (AI)** category includes digital tools using algorithms or artificial intelligence. The **digital mental healthcare** category includes digital mental health screening and telemedicine-based mental care. The **digital patient questionnaire (screening) tool** category includes electronic screening forms, computers for the collection of health behavior information, and other digital patient self-reported screening tools. The **online test ordering** category includes electronic test ordering systems and online diagnostic testing. The **teledermatology** category includes teledermatology and teledermatoscopy tools. The **self-management tools** category includes electronic patient-reported outcome tools and online patient self-regulation and support programs.	

### Study characteristics

Of the 208 selected articles, 81 were categorized as qualitative studies, 56 as quantitative descriptive studies, 24 as non-randomized studies, three as randomized controlled trials, and 44 as mixed methods studies, using the grouping method of the MMAT procedure. The majority of the studies used interviews (*n* = 108) or surveys (*n* = 90) to collect data. Focus groups were used in 38 of the included studies, and observational methods were used in 27 of the included studies.

More than half of the included studies (*n* = 125) were performed in North America: 110 studies originated from the United States and 15 were performed in Canada. Fifty-three of the included studies were performed in Europe, which mainly originated from the United Kingdom (*n* = 33). Further, eight studies were performed in Australia, eight studies in African countries, six studies in Middle Eastern countries, five studies in other Asian countries, and three studies in South America.

The majority of the included studies were published in 2013 (*n* = 27), 2018 (*n* = 30) and 2019 (*n* = 24), while only 12 and 14 of the included studies were published in respectively 2011 and 2010.

### Type of eHealth services

In total, the 208 included articles investigated 26 different types of eHealth services, which were categorized in 16 groups of eHealth services. Some studies covered eHealth services which could be categorized in more than one unique eHealth service category and some studies covered multiple eHealth services, outcomes or settings, resulting in a total of 304 effects of eHealth use on general practice workload that were assigned according to the method presented in the data analysis section. In total, in 63 of the included studies, more than one effect was found, while in 10 of these studies, a maximum of three effects were identified (i.e., ‘ + ’, ‘- ‘, and ‘0’).

With regard to the type of eHealth service being studied in the included studies, we identified that most of these investigated the impact of EHR use (*n* = 68) on the workload of general practice staff. Digital communication applications were investigated in 40 of the included studies. Furthermore, digital decision support services were described in 19 of the included studies, telemonitoring services in 16 studies, mHealth applications in 13 studies, and electronic prescribing applications also in 13 studies (see Table 1). A description of each eHealth category is also provided in Table 1.

Specification of the category ‘other eHealth services’ can be found in S Table 16 in Additional file 2. This category constitutes a group of eHealth services that were not described in any of the other included studies, and could consequently not be grouped in one eHealth service category. Table 1 also shows a breakdown of the (multiple) effects that were assigned to the studies’ main outcomes, i.e. the specific effects of the eHealth services use on general practice workload (based on the ‘ + ’/’- ‘/’0’ coding process described in the data analysis section). The division of the total number of 304 effects by type of eHealth service is similar to the division of the studies by type of eHealth service. Table 2 shows a similar breakdown of the studies analyzed and effects found, divided by type of study design.

**Table 2:** Studies by MMAT categorization and their assigned effects on general practice workload

Study design according to MMAT categorization	Number of references^*^	Number of effects assigned	+ ^a^ n (%)	-^b^ n (%)	0^c^ n (%)
Qualitative	81	109	72 (66)	31 (28)	6 (6)
Quantitative descriptive	56	73	35 (48)	27 (37)	11 (15)
Mixed method	44	67	30 (45)	26 (39)	11 (16)
Non-randomized	24	31	11 (35)	9 (29)	11 (35)
Randomized controlled trials	3	4	1 (25)	1 (25)	2 (50)
**Total**	**208**	**284**^**d**^	**149 (52)**	**94 (33)**	**41 (14)**

^*^The number of references is lower than the number of effects assigned because some studies reported more than one effect of eHealth service use on general practice workload (see the data analysis section)

^a^ Number of references reporting an increase in workload as a result of eHealth service use

^b^ Number of references reporting a reduction in workload as a result of eHealth service use

^c^ Number of references reporting no effect on workload as a result of eHealth service use

^d^ Because this analysis was focused on the MMAT-categorization and not on the type of eHealth service, this number is lower than the 304 effects assigned in Table 1

### Overall impact of eHealth services on practice workload

Tables 1 and 2 show that the impact of eHealth use or implementation on general practice workload differs between the type of eHealth service and type of study. Overall, a small majority of the studies found that eHealth use was associated with an increase in workload or a similar impact (e.g., lowered productivity or efficiency, more time-consuming). Based on the total number of effects assigned to the included studies, Table 1 shows that a slight majority (53%) indicates an increase in general practice workload as a result of eHealth use. In 33%, a reduction in workload, or a related outcome, was identified, and in the remaining 15%, there was no impact identified. These proportions are similar to the results in Table 2, based on a slightly lower number of assigned effects.

For the following eHealth service types it was found that a majority of the assigned effects showed an increase in general practice workload as a result of its use: EHR, digital decision support, telemonitoring, mHealth, and self-management tools. Only one eHealth service was identified for which a majority of the assigned effects showed a decrease in general practice workload because of its use: electronic prescribing. For the remaining eHealth service types, an ambiguous result was found. Furthermore, for almost all of the eHealth services, except for digital mental healthcare and digital patient questionnaire (screening) tools, the minority of the assigned effects indicated that eHealth use had no impact on general practice workload.

With regard to the MMAT study design categorization, we found for the qualitative studies that the majority of the assigned effects (66%) showed an increased general practice workload as a result of eHealth use, while in 28% a workload reduction effect was assigned, and in only 6% no impact was found (see Table 2). With regard to the other MMAT study designs, an ambiguous result was found. For the non-randomized studies, for example, 35% of the effects were assigned as having no impact, 35% as increasing general practice workload, and 29% as reducing general practice workload. With regard to the quantitative descriptive studies and mixed methods studies, the majority of assigned effects consisted of ‘ + ’ or ‘- ‘ indicators, while only a minority of the assigned effects was ‘0’. MMAT quality assessment scores for each study are presented in S Tables 1–16 in Additional file 2, so that the impact of eHealth services use on general practice workload can be considered in relation to the methodological quality of the studies. In general, the methodological quality of the included studies was rather good, especially for the qualitative studies. For mixed methods studies, in particular, methodological quality varied.

### EHealth services that more often led to an increased general practice workload

As mentioned before, EHR was one of the five eHealth service categories for which we found that the majority of the assigned effects (62%) reported an increase in general practice workload [18, 26–78]. Many of the studies focusing on this eHealth service reported that its use required extra time or work for physicians, leading to additional administration and registration or duplication of work [26, 31, 32, 34, 36, 37, 39, 45, 46, 48, 56, 59–72]. For example, Silva et al. [34] showed that EHR (functionality) implementation was associated with duplicate typing, rework and work overload [34]. About a quarter of the assigned effects (26%) of these studies indicated a reduction in workload or a related impact [16, 32, 33, 39, 40, 42, 45, 62–64, 66, 70, 72, 75, 79–87]. Several of these studies reported time savings for all, or specific general practice care staff. To illustrate this, a study by Kochendorfer et al. [16] showed that after five months of EHR use, residents and attending physicians reported daily time savings of 44 min [16]. Finally, only 11% of the assigned effects showed that some of these studies reported no impact on general practice workload, time, productivity, workflow, efficiency or the total number of contacts [28, 42, 60, 70, 80, 82, 88–91]. Furthermore, to illustrate, we assigned seventeen of the studies about EHR use and its impact on general practice workload with two or three effects [28, 32, 33, 39, 40, 42, 45, 60, 62–64, 66, 70, 72, 75, 80, 82]. One of these studies by Greiver et al. [63], for example, found that the implementation of an EHR led to an enormous increase in the amount of physicians’ time for data entry. On the other hand, some aspects of the EHR (i.e., prescription refills and consultation letters) made physicians more efficient after an initial decrease in efficiency. Physicians also thought that their administrative personnel were more efficient [63].

For digital decision support applications, slightly more than half of the assigned effects (52%) showed that its use increased general practice workload or had a related impact [92–104]. For example, Curry et al. (2011) reported that the time to use an electronic decision support tool was perceived as too long by physicians. Initially, 40% of the physicians reported that the decision support tool was disruptive in their workflow, which dropped to 16% when physicians gained more experience [94]. About one-third of the assigned effects (36%) reported a reduction in general practice workload or a related impact [93, 96, 98, 104–109]. To illustrate this, Barrett et al. [105] found that PCPs in their study felt that its use decreased time spent making clinical decisions, compared with the clinical tools they were used to utilize. Further, a minority of the assigned effects (12%) reported no impact on general practice workload as a result of digital decision support use [104, 109, 110].

With regard to telemonitoring services, a notable finding is that 76% of the effects indicated an increase in general practice workload [111–123]. For example, Webb et al. [123] found that managing a telemonitoring app led to significant additional work for support staff. The use of this app also added additional time to a consultation, and GPs felt rushed to address all related issues [123]. Only about one-fifth of the effects (18%) were assigned as reducing general practice workload [113, 120, 124, 125]. For example, a study by Davidson et al. (2019) found that the use of remote glucose monitoring saved time for both physicians and patients [125]. Finally, there was only one study by Kahalnik et al. [126] reporting no impact on general practice workload, indicating that providers reported a seamless integration of telemonitoring into the clinic’s existing workflows [126].

Furthermore, for mHealth services, the majority of the effects (68%) were assigned as increasing general practice workload [102, 113, 115, 118, 127–135]. For example, Diez-Canseco et al. [127] showed that time constraints and workload were reported as the main barriers to mHealth implementation, which involved doing more work within the same time [127]. On the other hand, a quarter of the effects (26%) was assigned as reducing general practice workload [113, 128, 131, 133, 135]. To illustrate, Mares et al. [131] showed in their study that some physicians reported that its use made less work for them [131]. Further, only one study by Schooley et al. [135] also reported that the vast majority of the providers in their study believed that it produced no negative impact on workload, indicating that no impact on workload was found [135].

Finally, the two included studies investigating self-management tools both found that these tools increased workload in general practice care [129, 136]. One of the two studies, performed by Poppe et al. (2018), reported that GPs identified the difficulty in integrating additional tasks into their daily workflow due to an overload of medical and administrative tasks as a result of the self-management tool. Furthermore, this study also reported that GPs mentioned that this tool sometimes required an additional consultation to motivate patients [136].

### EHealth services that more often led to a reduction in general practice workload

As mentioned before, electronic prescribing was the only eHealth service for which we found that a majority of the assigned effects (56%) reported a reduction in general practice workload or a related impact [59, 137–145]. To illustrate, Bulut et al. [139] found that a large share of family physicians indicated that the use of electronic prescriptions was speeding up the prescription process and saved time, as these prescriptions could be generated faster than manual prescriptions [139]. However, all of the other effects (44%) were assigned as reporting an increase in general practice workload [59, 137, 140, 142–144, 146–148]. For example, Devine et al. (2010) identified that electronic prescribing took longer than handwriting, according to PCPs [140]. For none of the studies, we found that the use of electronic prescribing had no impact on general practice workload.

### EHealth services with an ambiguous impact on general practice workload

For the remaining eHealth service categories, we did not find that a majority of the assigned effects indicated that its use increased or reduced general practice workload, or that its use had no impact on workload: digital communication, general health information technology, patient portal/patient online access, health information exchange (HIE), algorithms / AI, digital mental healthcare, digital patient questionnaire (screening) tools, online test ordering, and teledermatology.

With regard to digital communication services, the largest share of assigned effects (47%) showed an increase in workload or a related impact [30, 70, 149–173]. For example, Atherton et al. [149] showed that an increased workload was reported due to digital communication services use. Especially, video consultations were found to be time consuming to adopt [149]. On the other hand, several other studies found a reduction in general practice workload or a related impact because of digital communication use [47, 70, 151, 153, 156, 158, 161, 166, 168, 169, 174–180]. To illustrate this, Fagerlund et al. (2019) reported reduced phone load, increased efficiency, released time for medical assessments, and a reduction in the number of visits and phone contacts as a result of digital communication use in general practice [178]. Slightly fewer studies did not find an impact on general practice workload [70, 151, 154, 155, 160, 162, 166, 168, 175, 181–185]. For example, Edwards et al. (2017) reported in their study that any impact of e-consultations on general practice staff workload was likely to be negligible [182].

With regard to general health information technology services, the largest share of assigned effects (45%) showed a reduction in general practice workload [17, 70, 186–188]. For example, De Wilt et al. [188] found that almost all GPs in their study experienced that the eHealth services they used made their job more efficient and run more smoothly [188]. A slightly smaller proportion of the assigned effects indicated an increase in workload due to the use of general health information technology [51, 70, 189, 190]. One of these studies by Davidson et al. [189] reported that the integration of telehealth care data into the EHR of family practitioners led to concerns about the potential increased workload, particularly with regard to errors due to data overload [189]. Further, we identified two studies that reported no impact on workload [70, 191].

For patient portal/patient online access use, the largest share of assigned effects (40%) showed a workload reduction [172, 192–196], while 33% of these effects showed a workload increase [192, 194, 196–198]. About a quarter of the effects (27%) indicated that its use had no impact on workload [56, 192, 195, 196]. To illustrate, a study by Sorondo et al. [196] reported variable effects on practice workflow after the implementation of a patient portal: a reduction of time spent by medical assistants during office visits; no significant changes in the time spent by providers during the office visit; and an increase of time spent by physicians, care coordinators, and the office staff before the office visit were reported [196].

The included studies about HIE use reported either workload increases [199–203] or workload reductions [199–202, 204]. Mac McCullough (2014), for example, reported in their study that instances were found in which the used HIE system improved workflow within the studied primary care setting. However, instances were also found in which it appeared to hinder their workflow [200]. Most notably, none of the included studies found that HIE services had no impact on general practice workload.

Studies investigating eHealth services using algorithms or AI most often (44%) indicated that these lowered general practice workload [125, 205–207]. On the other hand, one-third of the assigned effects (33%) showed that this eHealth service increased general practice workload [205, 206, 208]. To illustrate, Mason et al. [208] found that GPs were concerned about the workload implications of assessment and care planning as a consequence of using a computer application (AnticiPal) with a search algorithm [208]. Furthermore, only two studies reported no impact on general practice workload as a result of its use [206, 207].

Studies investigating the association between digital mental healthcare applications and general practice workload more often (50% of the assigned effects) indicated that its use led to an increase in workload [127, 209, 210]. For example, Krog et al. [209] found that some of the GPs felt that the use of the digital mental healthcare tool was time-consuming [209]. One-third of the assigned effects (33%) reported no impact on workload [211, 212], while only one study showed that it was also associated with a reduction in general practice workload [210]. Montero-Marin et al. (2018), for example, found that there were no significant improvements in burnout among GPs as a result of using a blended web-based mindfulness program for GPs [212].

For digital patient questionnaire (screening) tools, we found that half of the assigned effects (50%) showed no impact on general practice workload because of their use [196, 213, 214]. For example, Paul et al. [214] reported that a majority of the GPs indicated that the implementation of a digital patient survey was not disruptive for their practice [214]. Further, one-third of the effects (33%) showed a workload reduction [196, 215], while only one study reported an increase in workload due to the use of a digital patient questionnaire tool [196].

With regard to the use of online test ordering, half of the effects (50%) indicated that its use led to a workload increase in general practice [216, 217]. The other half of the assigned effects (50%), however, showed a reduction in general practice workload [188, 217]. Whiting et al. [217], for example, found in their study that it was both associated with a workload increase and a workload reduction, depending on the phase of implementation. Initially, workload was adversely affected within the primary healthcare system. Finally, however, it was concluded that a systematic approach that aligns the use of blood tests to valid clinical questions eventually produced significant workload reductions [217].

Finally, for teledermatology services, an identical result was found. Two studies reported workload reductions because of its use [218, 219]. A similar amount of studies found the opposite [219, 220]. One of these studies, by McFarland et al. [219] reported both of these contrary outcomes, showing that providers reported an increase in workload as a result of teledermatology implementation, while 62% of the PCPs also agreed that teledermatology may save time [219].

### Other eHealth services had variable impacts on general practice workload

With regard to the eleven studies describing other eHealth services implementation and use in general practice care, the following eHealth services were investigated: smart devices, telemedical screening, a virtual quality improvement collaborative, an online education course, an electronic feedback system, technology-enabled academic detailing, social media, electronic standing orders, computer-based prototype consultation order templates, electronic medication refill system, and a computerized physician order entry tool [56, 221–230]. Impacts on general practice workload differed among these various other eHealth services and are shown in S Table 16 in Additional file 2.

## Discussion

### Principal findings

This is the first study to our knowledge to systematically investigate the impact of eHealth services use on workload in general practices in the pre-COVID-19 era. After assigning all 304 effects that were reported in the 208 included studies, we found that a small majority of evidence suggests that eHealth services in general lead to an increase in general practice workload. This is contrary to the main premises and expectation that eHealth will reduce workload in general practices. Still, this was indeed found in one-third of the cases. It should be noted, however, that a small part of the effects (15%) reported in the included studies indicated no impact of eHealth services use on this workload.

Looking at specific eHealth services, we see that the use of EHR, digital decision support, telemonitoring, mHealth, and self-management tools increase workload, while the use of electronic prescribing more often reduces workload in general practice care. For other specific eHealth service categories, including digital communication, general health information technology, patient portal/patient online access, HIE, digital mental healthcare, algorithms / AI, digital patient questionnaire (screening) tools, online test ordering, teledermatology, and other eHealth services, results are ambiguous.

It is not surprising that the relationship between the use of EHR and general practice workload has been investigated most often in comparison with other eHealth services, as this is one of the first digital innovations introduced into many general practices on a global level. With regard to its impact on general practice workload, a systematic literature review by Nguyen (2014) also found that a majority of the included studies in their review study reported an increased workload in healthcare settings due to its implementation compared to a smaller share of studies reporting a reduced workload in these settings. However, in this review, a larger share of included studies also reported increased administrative efficiency as a result of its implementation compared to studies reporting less administrative efficiency [231]. A more recent literature study by Budd [232] also reported that EHRs have become a significant contributor to physician burnout, generally due to factors such as poor usability, information overload, and EHR system alerts [232]. With regard to mHealth, this result is not in line with the findings of a systematic review by Gagnon et al. (2016), who reported that more studies indicated that mHealth saved healthcare professionals time when compared to studies reporting that mHealth may be time consuming and disruptive to the physicians’ workflow [233]. However, our results align with those from a scoping review by Addotey-Delove et al. [234], which reported increased workload among healthcare workers in the developing world due to mHealth implementation [234].

With regard to the use of patient online access services, our results are in line with the findings of the systematic review performed by De Lusignan et al. (2014), who also found a similar number of studies that reported either an increase or a reduction in general practice workload as a result of patient online access adoption [235]. However, a more recent systematic review by Tapuria et al. [236] investigating the impact of patient access to their EHR found several studies reporting increased consultation times after patients accessed their patient portal [236].

Further, Gagnon et al. [237] found variable results with regard to the implementation of electronic prescriptions, which differs slightly from what we found. They reported that its use was time consuming during the pre-implementation and transition phases, while time gains were found in post-implementation phase [237].

The main results of our review do, however, generally not match the promise of eHealth, indicating that it increases efficiency, and consequently reduces workload in general practices [1]. Our systematic overview makes it easier to determine which eHealth services more often lead to increased workload in general practice care, and in which situation or context. It may consequently support general practices or policymakers when making choices to solve health workforce challenges, including increasing workloads.

The results of this systematic review provide evidence that the use of eHealth in general practice care does not naturally lead to workload reductions and improved efficiencies within this healthcare sector. The variance in results suggests that factors other than the type of eHealth service also might contribute to its impact on general practice workload, including the setting, the characteristics (including digital skills and acceptance) of the healthcare professionals or patients, the usability, the design of the eHealth service, and the phase of implementation, which often differed between the included studies (see S Tables 1–16 in Additional file 2). Sittig et al. (2010) and Karsh et al. [238] highlighted the importance of considering these and other relevant socio-technical factors several years ago, when healthcare organizations were gradually implementing more eHealth, which generally impacted healthcare professionals’ workflow processes and workload [238, 239]. Furthermore, Ross et al. [240] categorized factors influencing the implementation of eHealth into the following categrories: the individual eHealth technology characteristics, the outer setting, the inner setting, characteristics of the individual health professionals, and the process of implementation [240]. More recently, Darley et al. [241] emphasized the importance of appropriate system design when implementing eHealth services (i.e., online consultations) in primary care. They found that primary care staff workload increased when online consultations were not integrated with existing software or workflows, while workloads decreased when sufficient resources were allocated [241]. Moreover, Smart et al. (2023) identified other relevant factors influencing variations in primary care staff workload due to online consultation implementation, including job role, practice context, and the form and rationale for implementation [242]. These socio-technical factors that may impact general practice workload or related outcomes (e.g., burnout, productivity, or consultation volume) as a result of eHealth service implementation were also described in several other studies, indicating the importance of considering these aspects when adopting new technologies [243–245]. With regard to our results, for example, it seems that more often an increase in general practice workload was identified in earlier stages of eHealth implementation or use, which is in line with the diffusion of innovation theory by Rogers et al. (2003) [246]. However, as not all of the included studies in our systematic review provided sufficient information about the phase or process of eHealth service implementation, we were not able to investigate this thoroughly. When eHealth adoption in general practice is encouraged by governmental bodies or implemented by general practices, it is important to consider these potential contributing factors and evaluate their impact on general practice organizations in order to prevent or reduce negative consequences, such as increased workloads [239].

### Strengths and Limitations and Future Research

Strengths of this study include its systematic and comparative approach, based on broad search terms and therefore a broad scope of eHealth applications. Likewise, we searched for different workload impacts in a large number of scientific databases, which resulted in a comprehensive overview of studies and an extensive coding of the effects they reported on the impact of eHealth on general practice workload. This comprehensive systematic approach could also be applied to other (sub)categorizations or frameworks of eHealth services, such as the OECD framework for telemedicine services, to investigate the impact of telemedicine services use on general practice workload [10]. Consequently, we also performed a short in-depth analysis, using the OECD framework for telemedicine services (consisting of three categories: store and forward telemedicine, interactive telemedicine, and telemonitoring), and identified 66 studies describing telemedicine services which investigated the impact of this service on workload in general practice. No clear association was found for store and forward telemedicine services and interactive telemedicine services either, as both workload increases and reductions were found as a result of telemedicine use or implementation. For telemonitoring services, however, it seems that the use of these services is more often associated with higher workloads among physicians, and is more often time-consuming compared to non-use.

However, some limitations of this review are also worth noting. First, a large share of the included studies were qualitative studies, mostly measuring subjective outcome measures, such as perceived workload. Only a few studies consisted of randomized controlled trials, which is generally seen as the gold standard for a study method in scientific research. Performing a good comparison between the various studies was accordingly difficult. Future work should consequently focus more on executing randomized controlled trials to investigate more accurately what the actual impact of eHealth service use or implementation is on workload in general practice. Importantly, looking at the different methodological designs, the workload increasing trend of specific eHealth services is consistent across studies, suggesting some robustness of our main findings.

Second, because of the heterogeneity of the study designs and study settings, it is difficult to compare the results of the included studies. Some studies had a large number of relevant study participants, while other studies had only a few participants or were unclear about the number of general practice staff. Consequently, it was not possible to weigh the results of the included studies according to the number of study participants. Furthermore, some studies were performed at the beginning of our study period (i.e., 2010), while others were executed just before the outbreak of the COVID-19 pandemic. In addition, the time frames sometimes differed considerably between the included studies, meaning that outcome measures might be dependent upon the moment when the use of a specific eHealth service was investigated. And finally, some eHealth services were already common practice at the beginning of our study period, while the use of other technologies was relatively new and controversial in general practice. Consequently, comparisons of the results between the examined eHealth services might be difficult and this may have led to publication bias, which might explain why certain eHealth services were studied more than others within the context of our study objective. This large heterogeneity prevented us from pooling studies and performing a meta-analysis. Another form of publication bias could be that studies that find no, or unexpected, effects are less likely to be published than studies that investigated significant effects in the expected direction, which could explain the result of our study that the smallest share of the effects of eHealth use in general practice was assigned as having no impact on workload [247]. Future research should consequently focus on homogenous subsets of studies in terms of eHealth services, study designs and study settings when performing new studies that focus on the relationship between eHealth use and general practice workload. In addition, it is recommended that future research should also focus on more in-depth and longitudinal research, taking into account the phase of implementation and the maturity of the eHealth service at the moment of performing the study.

Third, although most of the included studies clearly described the eHealth service that was investigated, it was sometimes difficult to categorize the studied eHealth services, as there is no unambiguous definition of eHealth [248]. For example, various terms are used for EHR, such as electronic medical record (EMR) or health management information system. In addition, sometimes different terms were used for similar eHealth services, including the terms telehealth and telemedicine. Future work should therefore also focus on defining global, unambiguous definitions for the various eHealth services that exist, making comparison between studies easier.

Fourth, we only included studies that were published in English, which might also explain why the majority of the included studies originated from Anglo-Saxon countries. Consequently, we might have missed important results from scientific papers performed in non-Anglo-Saxon countries, which were published in other languages. Future research could therefore focus on performing a similar study, including studies that were published in other major languages than English, for example in Spanish or Chinese language. Results could then be compared with our study results.

Fifth, our eHealth categorization showed that only little research has been performed on some of the eHealth service types in relation to their impact on general practice workload, such as self-management tools, teledermatology, and online test ordering (see Table 1). Future work should consequently also focus more on investigating the impact of these less explored services, while less additional research on eHealth services including EHR and digital communication services is needed, as the impact of these services on general practice workload has already been investigated more often.

Finally, in our study we focused on the period 2010 until the start of the COVID-19 pandemic, making these results less comparable to the period during the COVID-19 pandemic, which radically changed the organization of healthcare, including general practice care. As this pandemic has come to an end, we think that our study results can be of added value for current general practice care. However, future research could investigate what influence the pandemic has had on the use of eHealth on workload in general practices.

## Conclusions

Our systematic review of 208 studies shows that a small majority of the 304 effects these studies reported, rather indicate an increase instead of a reduction in general practice workload because of eHealth implementation and use.

Various eHealth services were identified through our systematic review and for several of these services, we found that a larger share of the effects indicated workload increases. Specifically, this was observed for the use of telemonitoring, mHealth and EHR applications. However, for one other eHealth service (i.e., electronic prescribing), a small majority of the effects indicated a reduction in general practice workload. These findings are not consistent with the promise of eHealth to increase efficiency in healthcare organizations. However, the impact of eHealth services use on general practice workload was found to be ambiguous, making it difficult to draw a definitive conclusion, which underscores the need for further explanatory research.

Furthermore, various factors can influence the impact of eHealth use on workload and routine workflow processes in general practice, including the setting, the characteristics of the healthcare professionals or patients, usability, system design, and the phase of implementation. These factors should be considered when eHealth is encouraged by governmental bodies or adopted in general practice.

## Supplementary Information

Additional file 1. Search strings and search strategy results, April 21st, 2020. Description of data: Overview of the included search databases (S table 1), the number of resulting references, and the search strings for each of the included search databases.

Additional file 2. Overview of included studies with results about the impact of eHealth use on general practice workload. Description of data: Overview of the main characteristics and results of the included studies in the systematic review study, including MMAT quality assessment results (S tables 1-16).

Additional file 3. PRISMA-P 2015 checklist. Description of data: PRISMA-P (Preferred Reporting Items for Systematic review and Meta-Analysis Protocols) 2015 checklist: recommended items to address in a systematic review protocol (S tables 1).

## Data Availability

The datasets used and/or analyzed during the current study are available from the corresponding author on reasonable request.

## Abbreviations

CPOE: Computerized physician order entry
EHR: Electronic Health Record (Electronic Medical Record (EMR) is also considered as EHR)
GP: General practitioner
HIE: Health information exchange
HIT: Health information technology
MA: Medical Assistant
MD: Medical Doctor
MeSH: Medical Subject Headings
NP: Nurse Practitioner
OM: Office Manager
PA: Physician Assistant
PN: Practice Nurse
PCP: Primary care physician
RCT: Randomized controlled trial
UAE: United Arab Emirates
UK: United Kingdom
US: United States of America
