# Supplementary material for: The impact of eHealth use on general practice workload in the pre-COVID-19 era: a systematic review
Source: BMC Health Serv Res. 2024 Sep 19;24:1099. doi: 10.1186/s12913-024-11524-9 (PMC11414290; doi:10.1186/s12913-024-11524-9)
Supplement: Supplementary file 1 — Additional file 1. Search strings and search strategy results, April 21st, 2020. Description of data: Overview of the included search databases (S table 1), the number of resulting references, and the search strings for each of the included search databases. [file 12913_2024_11524_MOESM1_ESM.docx]

## Additional file 1 – Search strings and search strategy results, April 21^st^, 2020

### Overview of the included search databases and the number of resulting references

**S Table 1** Overview of databases and the number of resulting references after de-duplication

| Database searched | Source | Years of coverage | Number of references | Number of references after de-duplication |
| --- | --- | --- | --- | --- |
| CINAHL | EBSCO | 1982 - Present | 2,852 | 1,291 |
| Cochrane Central Register of Controlled Trials | Wiley | 1992 - Present | 1,003 | 701 |
| Embase | Embase.com | 1971 - Present | 2,928 | 2,879 |
| IEEE Xplore | ieeexplore.ieee.org |  | 13 | 12 |
| Medline ALL | Ovid | 1946 - Present | 2,196 | 488 |
| PsycINFO | Ovid | 1806 - Present | 424 | 86 |
| Web of Science Core Collection | Web of Knowledge | 1975 - Present | 2,750 | 1,192 |
| Other sources: Google Scholar | | | 200 | 178 |
| Total | | | **12,366** | **6,827** |

### Search strings for each of the included databases

**Embase – 2,928 references**

('telehealth'/exp OR 'telecommunication'/de OR 'teleconsultation'/de OR 'mobile application'/exp OR 'microcomputer'/de OR 'web-based intervention'/de OR 'Internet'/exp OR 'internet protocol'/de OR mhealth/de OR 'wearable computer'/exp OR 'mobile phone'/exp OR 'social media'/de OR 'electronic medical record system'/de OR 'computer system'/de OR 'electronic health record'/exp OR 'telecommuting'/de OR 'text messaging'/de OR 'personal digital assistant'/exp OR 'decision support system'/exp OR ('attitude to computers'/de AND ('health personnel attitude'/de OR 'physician attitude'/de)) OR (portal OR portals OR digitize* OR texting OR messaging OR text-messag* OR ((digital*) NEAR/3 (assistant*)) OR ((decision* OR decid*) NEAR/6 (support* OR assist* OR rule*) NEAR/6 (system)) OR EHR OR EHRS OR video* OR telecommunic* OR telehealth* OR telepath* OR telecardio* OR telepatho* OR telepsych* OR therap* OR teleconsult* OR telecare* OR telemonitor* OR telerehabilitat* OR telemetr* OR ((tele OR e OR m OR mobile* OR online* OR electronic* OR computer* OR wireless OR portable OR remote*) NEAR/3 (care OR consult* OR communic* OR health* OR medicin* OR medical* OR nurs* OR consult* OR diagnos* OR monitor* OR cardio* OR patho* OR psych* OR derma* OR surgic* OR radiolog* OR system* OR based OR decision* OR decid* OR rehabilitat* OR conferenc*)) OR ((mobile OR phone) NEAR/3 (application* OR tool*)) OR mobile-phone* OR app OR apps OR smartphone* OR smart-phone* OR smartwatch* OR smart-watch* OR smart-glass* OR smartglass* OR microcomputer* OR micro-computer* OR ((web-based OR webbased OR internet OR website* OR web-site*) NEAR/3 (app OR application* OR tool OR system OR systems OR monitoring)) OR device OR social-media OR facebook OR twitter OR Instagram OR mobile-technolog* OR (health NEAR/3 information NEAR/3 (technolog* OR innovation*)) OR wearable*):ab,ti,kw) **AND** ('workplace'/de OR 'workload'/de OR 'working time'/de OR 'work experience'/de OR 'work environment'/de OR 'work engagement'/de OR 'quality of working life'/de OR 'productivity'/de OR 'presenteeism'/de OR 'job stress'/exp OR 'job satisfaction'/de OR 'job performance' OR 'job experience'/de OR 'absenteeism'/de OR 'burnout'/exp OR 'job analysis'/de OR 'workflow'/de OR 'organizational efficiency'/de OR 'process optimization'/de OR ('attitude to computers'/de AND ('health personnel attitude'/de OR 'physician attitude'/de)) OR (((time) NEAR/3 (saving)) OR OLE OR presenteeism OR absenteeism OR workflow* OR work-flow* OR productivit* OR workload* OR workplace OR worklocation* OR work-time* OR ((working-life*) NEAR/3 (quality*)) OR ((work OR job* OR organization* OR organisation* OR labor OR occupat* OR staff) NEAR/3 (place* OR location* OR site* OR load* OR participat* OR stress* OR satisf* OR dissatisf* OR offload* OR efficien* OR effectiv* OR experienc* OR overtime OR performance* OR fatigue* OR engag* OR environment* OR climat* OR planning OR ill OR illness* OR sick*)) OR burnout* OR burn-out* OR revenue* OR ((task*) NEAR/3 (shift* OR substitut*)) OR ((administrat* OR registrat*) NEAR/3 (burden))):ab,ti,kw) **AND** ('general practice'/de OR 'primary health care'/exp OR 'general practitioner'/de OR 'family medicine'/de OR (((primary OR family OR general OR first-line) NEAR/3 (care OR pract* OR medicine* OR physician* OR doctor*)) OR gp OR gps):ab,ti,kw) *AND [2005-2021]/py NOT ((animal/exp OR animal*:de OR nonhuman/de) NOT ('human'/exp)) AND [english]/lim NOT ([Conference Abstract]/lim)*

**Medline – 2,196 references** *(limit 2005-2021 in database)*

(exp Telemedicine/ OR Telecommunications/ OR exp Telemetry/ OR Mobile Applications/ OR exp Microcomputers/ OR exp Cell Phone/ OR exp Medical Records Systems, Computerized/ OR exp Computer Systems/ OR (Attitude to Computers/ AND (Attitude of Health Personnel/)) OR (portal OR portals OR digitize* OR texting OR messaging OR text-messag* OR ((digital*) ADJ3 (assistant*)) OR ((decision* OR decid*) ADJ6 (support* OR assist* OR rule*) ADJ6 (system)) OR EHR OR EHRS OR video* OR telecommunic* OR telehealth* OR telepath* OR telecardio* OR telepatho* OR telepsych* OR therap* OR teleconsult* OR telecare* OR telemonitor* OR telerehabilitat* OR telemetr* OR ((tele OR e OR m OR mobile* OR online* OR electronic* OR computer* OR wireless OR portable OR remote*) ADJ3 (care OR consult* OR communic* OR health* OR medicin* OR medical* OR nurs* OR consult* OR diagnos* OR monitor* OR cardio* OR patho* OR psych* OR derma* OR surgic* OR radiolog* OR system* OR based OR decision* OR decid* OR rehabilitat* OR conferenc*)) OR ((mobile OR phone) ADJ3 (application* OR tool*)) OR mobile-phone* OR app OR apps OR smartphone* OR smart-phone* OR smartwatch* OR smart-watch* OR smart-glass* OR smartglass* OR microcomputer* OR micro-computer* OR ((web-based OR webbased OR internet OR website* OR web-site*) ADJ3 (app OR application* OR tool OR system OR systems OR monitoring)) OR device OR social-media OR facebook OR twitter OR Instagram OR mobile-technolog* OR (health ADJ3 information ADJ3 (technolog* OR innovation*)) OR wearable*).ab,ti.) **AND** (Workplace/ OR Workload/ OR Work Engagement/ OR Efficiency, Organizational/ OR Presenteeism/ OR exp Occupational Stress/ OR Job Satisfaction/ OR Work Performance/ OR Absenteeism/ OR Workflow/ OR (Attitude to Computers/ AND (Attitude of Health Personnel/)) OR (((time) ADJ3 (saving)) OR OLE OR presenteeism OR absenteeism OR workflow* OR work-flow* OR productivit* OR workload* OR workplace OR worklocation* OR work-time* OR ((working-life*) ADJ3 (quality*)) OR ((work OR job* OR organization* OR organisation* OR labor OR occupat* OR staff) ADJ3 (place* OR location* OR site* OR load* OR participat* OR stress* OR satisf* OR dissatisf* OR offload* OR efficien* OR effectiv* OR experienc* OR overtime OR performance* OR fatigue* OR engag* OR environment* OR climat* OR planning OR ill OR illness* OR sick*)) OR burnout* OR burn-out* OR revenue* OR ((task*) ADJ3 (shift* OR substitut*)) OR ((administrat* OR registrat*) ADJ3 (burden))).ab,ti.) **AND** (exp General Practice/ OR Primary Health Care/ OR General Practitioners/ OR (((primary OR family OR general OR first-line) ADJ3 (care OR pract* OR medicine* OR physician* OR doctor*)) OR gp OR gps).ab,ti.) NOT ((exp animal/) NOT (human/)) AND english.la. NOT (news OR congres* OR abstract* OR book* OR chapter* OR dissertation abstract*).pt.

**Cochrane – 1,003 references** *(2005-2021 manually in EndNote)*

((portal OR portals OR digitize* OR texting OR messaging OR text-messag* OR ((digital*) NEAR/3 (assistant*)) OR ((decision* OR decid*) NEAR/6 (support* OR assist* OR rule*) NEAR/6 (system)) OR EHR OR EHRS OR video* OR telecommunic* OR telehealth* OR telepath* OR telecardio* OR telepatho* OR telepsych* OR therap* OR teleconsult* OR telecare* OR telemonitor* OR telerehabilitat* OR telemetr* OR ((tele OR e OR m OR mobile* OR online* OR electronic* OR computer* OR wireless OR portable OR remote*) NEAR/3 (care OR consult* OR communic* OR health* OR medicin* OR medical* OR nurs* OR consult* OR diagnos* OR monitor* OR cardio* OR patho* OR psych* OR derma* OR surgic* OR radiolog* OR system* OR based OR decision* OR decid* OR rehabilitat* OR conferenc*)) OR ((mobile OR phone) NEAR/3 (application* OR tool*)) OR mobile-phone* OR app OR apps OR smartphone* OR smart-phone* OR smartwatch* OR smart-watch* OR smart-glass* OR smartglass* OR microcomputer* OR micro-computer* OR ((web-based OR webbased OR internet OR website* OR web-site*) NEAR/3 (app OR application* OR tool OR system OR systems OR monitoring)) OR device OR social-media OR facebook OR twitter OR Instagram OR mobile-technolog* OR (health NEAR/3 information NEAR/3 (technolog* OR innovation*)) OR wearable*):ab,ti,kw) **AND** ((((time) NEAR/3 (saving)) OR OLE OR presenteeism OR absenteeism OR workflow* OR work-flow* OR productivit* OR workload* OR workplace OR worklocation* OR work-time* OR ((working-life*) NEAR/3 (quality*)) OR ((work OR job* OR organization* OR organisation* OR labor OR occupat* OR staff) NEAR/3 (place* OR location* OR site* OR load* OR participat* OR stress* OR satisf* OR dissatisf* OR offload* OR efficien* OR effectiv* OR experienc* OR overtime OR performance* OR fatigue* OR engag* OR environment* OR climat* OR planning OR ill OR illness* OR sick*)) OR burnout* OR burn-out* OR revenue* OR ((task*) NEAR/3 (shift* OR substitut*)) OR ((administrat* OR registrat*) NEAR/3 (burden))):ab,ti,kw) **AND** ((((primary OR family OR general OR first-line) NEAR/3 (care OR pract* OR medicine* OR physician* OR doctor*)) OR gp OR gps):ab,ti,kw)

**Web of Science – 2,750 references** *(limit 2005-2021 in database)*

TS=(((portal OR portals OR digitize* OR texting OR messaging OR text-messag* OR ((digital*) NEAR/2 (assistant*)) OR ((decision* OR decid*) NEAR/5 (support* OR assist* OR rule*) NEAR/5 (system)) OR EHR OR EHRS OR video* OR telecommunic* OR telehealth* OR telepath* OR telecardio* OR telepatho* OR telepsych* OR therap* OR teleconsult* OR telecare* OR telemonitor* OR telerehabilitat* OR telemetr* OR ((tele OR e OR m OR mobile* OR online* OR electronic* OR computer* OR wireless OR portable OR remote*) NEAR/2 (care OR consult* OR communic* OR health* OR medicin* OR medical* OR nurs* OR consult* OR diagnos* OR monitor* OR cardio* OR patho* OR psych* OR derma* OR surgic* OR radiolog* OR system* OR based OR decision* OR decid* OR rehabilitat* OR conferenc*)) OR ((mobile OR phone) NEAR/2 (application* OR tool*)) OR mobile-phone* OR app OR apps OR smartphone* OR smart-phone* OR smartwatch* OR smart-watch* OR smart-glass* OR smartglass* OR microcomputer* OR micro-computer* OR ((web-based OR webbased OR internet OR website* OR web-site*) NEAR/2 (app OR application* OR tool OR system OR systems OR monitoring)) OR device OR social-media OR facebook OR twitter OR Instagram OR mobile-technolog* OR (health NEAR/2 information NEAR/2 (technolog* OR innovation*)) OR wearable*)) AND ((((time) NEAR/2 (saving)) OR OLE OR presenteeism OR absenteeism OR workflow* OR work-flow* OR productivit* OR workload* OR workplace OR worklocation* OR work-time* OR ((working-life*) NEAR/2 (quality*)) OR ((work OR job* OR organization* OR organisation* OR labor OR occupat* OR staff) NEAR/2 (place* OR location* OR site* OR load* OR participat* OR stress* OR satisf* OR dissatisf* OR offload* OR efficien* OR effectiv* OR experienc* OR overtime OR performance* OR fatigue* OR engag* OR environment* OR climat* OR planning OR ill OR illness* OR sick*)) OR burnout* OR burn-out* OR revenue* OR ((task*) NEAR/2 (shift* OR substitut*)) OR ((administrat* OR registrat*) NEAR/2 (burden)))) AND ((((primary OR family OR general OR first-line) NEAR/2 (care OR pract* OR medicine* OR physician* OR doctor*)) OR gp OR gps)) NOT ((animal* OR rat OR rats OR mouse OR mice OR murine OR dog OR dogs OR canine OR cat OR cats OR feline OR rabbit OR cow OR cows OR bovine OR rodent* OR sheep OR ovine OR pig OR swine OR porcine OR veterinar* OR chick* OR zebrafish* OR baboon* OR nonhuman* OR primate* OR cattle* OR goose OR geese OR duck OR macaque* OR avian* OR bird* OR fish*) NOT (human* OR patient* OR women OR woman OR men OR man))) AND DT=(Article OR Review) AND LA=(English)

**PsycINFO – 424 references** *(limit 2005-2021 in database)*

(exp Telemedicine/ OR exp Wireless Technologies/ OR Mobile Applications/ OR exp Microcomputers/ OR exp Mobile Phones/ OR exp Electronic Health Records/ OR exp Computer Applications/ OR (portal OR portals OR digitize* OR texting OR messaging OR text-messag* OR ((digital*) ADJ3 (assistant*)) OR ((decision* OR decid*) ADJ6 (support* OR assist* OR rule*) ADJ6 (system)) OR EHR OR EHRS OR video* OR telecommunic* OR telehealth* OR telepath* OR telecardio* OR telepatho* OR telepsych* OR therap* OR teleconsult* OR telecare* OR telemonitor* OR telerehabilitat* OR telemetr* OR ((tele OR e OR m OR mobile* OR online* OR electronic* OR computer* OR wireless OR portable OR remote*) ADJ3 (care OR consult* OR communic* OR health* OR medicin* OR medical* OR nurs* OR consult* OR diagnos* OR monitor* OR cardio* OR patho* OR psych* OR derma* OR surgic* OR radiolog* OR system* OR based OR decision* OR decid* OR rehabilitat* OR conferenc*)) OR ((mobile OR phone) ADJ3 (application* OR tool*)) OR mobile-phone* OR app OR apps OR smartphone* OR smart-phone* OR smartwatch* OR smart-watch* OR smart-glass* OR smartglass* OR microcomputer* OR micro-computer* OR ((web-based OR webbased OR internet OR website* OR web-site*) ADJ3 (app OR application* OR tool OR system OR systems OR monitoring)) OR device OR social-media OR facebook OR twitter OR Instagram OR mobile-technolog* OR (health ADJ3 information ADJ3 (technolog* OR innovation*)) OR wearable*).ab,ti.) **AND** (Workload/ OR Employee Engagement/ OR Employee Efficiency/ OR Presenteeism/ OR exp Occupational Stress/ OR Job Satisfaction/ OR Work Performance/ OR Employee Productivity/ OR Job Performance/ OR Employee Absenteeism/ OR (((time) ADJ3 (saving)) OR OLE OR presenteeism OR absenteeism OR workflow* OR work-flow* OR productivit* OR workload* OR workplace OR worklocation* OR work-time* OR ((working-life*) ADJ3 (quality*)) OR ((work OR job* OR organization* OR organisation* OR labor OR occupat* OR staff) ADJ3 (place* OR location* OR site* OR load* OR participat* OR stress* OR satisf* OR dissatisf* OR offload* OR efficien* OR effectiv* OR experienc* OR overtime OR performance* OR fatigue* OR engag* OR environment* OR climat* OR planning OR ill OR illness* OR sick*)) OR burnout* OR burn-out* OR revenue* OR ((task*) ADJ3 (shift* OR substitut*)) OR ((administrat* OR registrat*) ADJ3 (burden))).ab,ti.) **AND** (exp General Practice/ OR Primary Health Care/ OR General Practitioners/ OR Family Physicians/ OR (((primary OR family OR general OR first-line) ADJ3 (care OR pract* OR medicine* OR physician* OR doctor*)) OR gp OR gps).ab,ti.) NOT ((animal.po. OR exp animals/) NOT human.po.) AND english.la. NOT (news OR congres* OR abstract* OR book* OR chapter* OR dissertation abstract*).pt.

**Cinahl – 2852 references** *(limit 2005-2021 in database)*

(MH Telemedicine+ OR MH Telecommunications+ OR MH Telemetry+ OR MH Mobile Applications+ OR MH Microcomputers+ OR MH Cellular Phone+ OR MH Electronic Health Records+ OR MH Computer Systems+ OR (MH Attitude to Computers+ AND (Attitude of Health Personnel+)) OR (**(TI(**portal OR portals OR digitize* OR texting OR messaging OR text-messag* OR ((digital*) N2 (assistant*)) OR ((decision* OR decid*) N5 (support* OR assist* OR rule*) N5 (system)) OR EHR OR EHRS OR video* OR telecommunic* OR telehealth* OR telepath* OR telecardio* OR telepatho* OR telepsych* OR therap* OR teleconsult* OR telecare* OR telemonitor* OR telerehabilitat* OR telemetr* OR ((tele OR e OR m OR mobile* OR online* OR electronic* OR computer* OR wireless OR portable OR remote*) N2 (care OR consult* OR communic* OR health* OR medicin* OR medical* OR nurs* OR consult* OR diagnos* OR monitor* OR cardio* OR patho* OR psych* OR derma* OR surgic* OR radiolog* OR system* OR based OR decision* OR decid* OR rehabilitat* OR conferenc*)) OR ((mobile OR phone) N2 (application* OR tool*)) OR mobile-phone* OR app OR apps OR smartphone* OR smart-phone* OR smartwatch* OR smart-watch* OR smart-glass* OR smartglass* OR microcomputer* OR micro-computer* OR ((web-based OR webbased OR internet OR website* OR web-site*) N2 (app OR application* OR tool OR system OR systems OR monitoring)) OR device OR social-media OR facebook OR twitter OR Instagram OR mobile-technolog* OR (health N2 information N2 (technolog* OR innovation*)) OR wearable*))**) OR AB(**portal OR portals OR digitize* OR texting OR messaging OR text-messag* OR ((digital*) N2 (assistant*)) OR ((decision* OR decid*) N5 (support* OR assist* OR rule*) N5 (system)) OR EHR OR EHRS OR video* OR telecommunic* OR telehealth* OR telepath* OR telecardio* OR telepatho* OR telepsych* OR therap* OR teleconsult* OR telecare* OR telemonitor* OR telerehabilitat* OR telemetr* OR ((tele OR e OR m OR mobile* OR online* OR electronic* OR computer* OR wireless OR portable OR remote*) N2 (care OR consult* OR communic* OR health* OR medicin* OR medical* OR nurs* OR consult* OR diagnos* OR monitor* OR cardio* OR patho* OR psych* OR derma* OR surgic* OR radiolog* OR system* OR based OR decision* OR decid* OR rehabilitat* OR conferenc*)) OR ((mobile OR phone) N2 (application* OR tool*)) OR mobile-phone* OR app OR apps OR smartphone* OR smart-phone* OR smartwatch* OR smart-watch* OR smart-glass* OR smartglass* OR microcomputer* OR micro-computer* OR ((web-based OR webbased OR internet OR website* OR web-site*) N2 (app OR application* OR tool OR system OR systems OR monitoring)) OR device OR social-media OR facebook OR twitter OR Instagram OR mobile-technolog* OR (health N2 information N2 (technolog* OR innovation*)) OR wearable*)**))**) AND (MH Work Environment+ OR MH Workload+ OR MH Work Engagement+ OR MH Organizational Efficiency+ OR MH Presenteeism+ OR MH Stress, Occupational+ OR MH Job Satisfaction+ OR MH Job Performance+ OR MH Absenteeism+ OR MH Workflow+ OR (MH Attitude to Computers+ AND (MH Attitude of Health Personnel+)) OR (**(TI(**((time) N2 (saving)) OR OLE OR presenteeism OR absenteeism OR workflow* OR work-flow* OR productivit* OR workload* OR workplace OR worklocation* OR work-time* OR ((working-life*) N2 (quality*)) OR ((work OR job* OR organization* OR organisation* OR labor OR occupat* OR staff) N2 (place* OR location* OR site* OR load* OR participat* OR stress* OR satisf* OR dissatisf* OR offload* OR efficien* OR effectiv* OR experienc* OR overtime OR performance* OR fatigue* OR engag* OR environment* OR climat* OR planning OR ill OR illness* OR sick*)) OR burnout* OR burn-out* OR revenue* OR ((task*) N2 (shift* OR substitut*)) OR ((administrat* OR registrat*) N2 (burden)))**) OR AB(**((time) N2 (saving)) OR OLE OR presenteeism OR absenteeism OR workflow* OR work-flow* OR productivit* OR workload* OR workplace OR worklocation* OR work-time* OR ((working-life*) N2 (quality*)) OR ((work OR job* OR organization* OR organisation* OR labor OR occupat* OR staff) N2 (place* OR location* OR site* OR load* OR participat* OR stress* OR satisf* OR dissatisf* OR offload* OR efficien* OR effectiv* OR experienc* OR overtime OR performance* OR fatigue* OR engag* OR environment* OR climat* OR planning OR ill OR illness* OR sick*)) OR burnout* OR burn-out* OR revenue* OR ((task*) N2 (shift* OR substitut*)) OR ((administrat* OR registrat*) N2 (burden)))**)))** AND (MH Primary Health Care+ OR MH Physicians, Family+ OR (**(TI(**((primary OR family OR general OR first-line) N2 (care OR pract* OR medicine* OR physician* OR doctor*)) OR gp OR gps)**) OR AB(**((primary OR family OR general OR first-line) N2 (care OR pract* OR medicine* OR physician* OR doctor*)) OR gp OR gps)**)))**

**IEEE Xplore – 13 references** *(limit 2005-2021 in database)*

((("Full Text & Metadata":telehealth OR telecommunication OR teleconsultation OR mhealth OR ehealth ) AND "Full Text & Metadata":workplace OR workload OR productivity OR work-stress OR work-efficiency OR work-quality OR work-satisfaction ) AND "Full Text & Metadata":primary-care OR primary-healthcare OR general-practioner)

**Google Scholar – 200 references** (random top-200, no data limit)

telehealth|telecommunication|teleconsultation|mhealth|ehealth workplace|workload|productivity|"work|job stress|efficiency|quality|satisfaction" "primary|family|general care|practioner|medicine"
