# Supplementary material for: The impact of eHealth use on general practice workload in the pre-COVID-19 era: a systematic review
Source: BMC Health Serv Res. 2024 Sep 19;24:1099. doi: 10.1186/s12913-024-11524-9 (PMC11414290; doi:10.1186/s12913-024-11524-9)
Supplement: Supplementary file 2 — Additional file 2. Overview of included studies with results about the impact of eHealth use on general practice workload. Description of data: Overview of the main characteristics and results of the included studies in the systematic review study, including MMAT quality assessment results (S tables 1-16). [file 12913_2024_11524_MOESM2_ESM.docx]

### Additional file 2 – Overview of included studies with results about the impact of eHealth use on general practice workload

**S table 1** Overview main characteristics of included studies - EHR

| Study ID | Methods / Design | Participants | Country | Intervention | Outcome measure(s) and key result(s) | Notes |
| --- | --- | --- | --- | --- | --- | --- |
| Adler et al (18), 2015  MMAT score: 5 | Survey | 305 family physicians | US | EHR | More users disagreed that their new EHR improved productivity than agreed. | A similar share of users disagreed or agreed that the EHR system is faster than the former system. |
| Adler-Milstein et al (85), 2013  MMAT score: 3 | Observations | Clinical (support) staff working in 42 primary care and internal medicine practices | US | EHR | Greater EHR use was independently associated with higher levels of productivity. | EHR use had a positive impact on productivity in large practices, but a negative joint impact on productivity in small practices. |
| Adler-Milstein et al (49), 2020  MMAT score: 4 | Mixed methods:  1. survey  2. EHR use measures  3. health system data | 122 PCPs from 10 comprehensive primary care practices in a large academic health system | US | EHR | EHRs is seen as a contributor to clinician burnout, and data suggest a relationship between EHR use and burnout. | Study results suggest that EHR workload primarily impacts exhaustion rather than cynicism. Only 17% self-reported optimal or modest burden of time spent on the EHR at home. |
| Agana et al (50), 2017  MMAT score: 5 | Focus group | 26 family physicians from 6 academic family medicine clinics | US | EHR (functionalities) | Family physicians did not feel they had the time to complete administrative tasks of patient care in the EHR.  Time associated with administrative activities and the EHR were associated with negative comments by physicians compared to other themes associated with physician burnout. |  |
| Al Alawi et al (59), 2014  MMAT score: 5 | Focus group | 23 PCPs | UAE | EHR, including electronic prescription | PCPs reported that the EHR documentation was time-consuming, especially during the start of implementation. | PCPs believed that the increased patient waiting time was mainly in the registration and nursing assessment, as they had to do a lot of things. |
| Ariza et al (77), 2015  MMAT score: 4 | Survey | 67 GPs | UK | EHR (functionalities) | Four EHR tasks imposed a higher mental workload on GPs.  The workload score found in this study appears to be low, compared to other studies. | Significant differences among the task workload scores were found, but the aggregate workload score was not different among EHR systems. |
| Asan et al (79), 2015  MMAT score: 2 | (Video) Observations | 6 PCPs and 18 patients | US | EHR | Integration of the EHR system into PCPs’ work creates highly variable workflows, and EHR influences workflow. | PCPs were classified into three EHR interaction styles. All PCPs used the same EHR system. |
| Babbott et al (78), 2014  MMAT score: 4 | Survey | 379 PCPs and 92 clinic managers from 92 primary care clinics | US | EHR (functionalities) | PCPs using an EHR with a moderate number of functions report more stress than PCPs with fewer EHR functions.  Time pressure was more related to adverse PCP’s stress and burnout in the high EHR function cluster. | There was no clear association between years since EHR implementation and work stress. |
| Bae et al (60), 2016  MMAT score: 5 | Survey | 1,470 PCPs (including GPs and family practitioners) | US | EHR | Adoption of EHR among PCPs was associated with 1.5 extra hours spent on patient face time per week for each PCP, with no change in the overall number of visits per week.  EHR does not seem to free up enough time in the week for PCPs to see additional patients. | For one subpopulation of PCPs (age > 61), EHR use did seem to free up time for PCPs to take on additional patients. They expanded visits per week by 30-40% when using an EHR. |
| Ballout et al (81), 2018  MMAT score: 2 | EHR log data | 121 primary healthcare centers, serving 3 million Palestine refugees. | Jordan, Syria, Lebanon, West Bank and Gaza | EHR | The number of physician’s daily consultations was reduced from 104 to 85. Almost all physicians indicated time savings and efficiency related to the eHealth system.  It resulted in the reduction of the physician’s time to hand-write the prescriptions. |  |
| Bhargava et al (88), 2014  MMAT score: 4 | Panel data:  1. productivity  2. observations 3. interviews  4. survey | 87 PCPs from 12 primary care clinics of an academic hospital. | US | EHR | EHR systems do not produce the kind of productivity gain that could lead to substantial savings in healthcare; at the same time, EHRs do not cause a major productivity loss on a sustained basis, as many physicians fear. | Results suggest that generic EHRs may not be suited equally for all the physician specialties. |
| Bhavsar et al (41), 2014  MMAT score: 4 | Survey | 426 primary care practice groups, representing 1,066 individual practice sites. | US | EHR | A majority of the practices without an EHR system perceived productivity disruption because of EHR investment.  Especially at the start of the EHR implementation phase, most of the practices expected a decrease in productivity. | CITIA-SC practice sites faced similar barriers to EHR implementation as non-CITIA-SC participants, including productivity disruption. |
| Bouamrane et al (61), 2013  MMAT score: 5 | Interviews and focus group | 25 GPs | UK | EHR | EHR is administratively cumbersome and/or not sufficiently flexible to support workflows. | The EHR system was on average over 5 and a half years in use (range: 1 to 22 years). |
| Brown Jr et al (40), 2014  MMAT score: 4 | 1. surveys  2. clinical logs 3. spread-sheets in mixed electronic and paper formats | 1,000 patients, 99 clinical staff members (pre-implementation), 155 clinical staff members (post-implementation) | US | EHR | Productivity declined for counselling staff and for medical services staff. Productivity increased for case manager staff.  Pre-implementation: more counselling visits and primary medical care visits compared to post-implementation period. However, less case manager HIV counselling visits occurred. |  |
| Cajander et al (26), 2018  MMAT score: 5 | Interviews | 7 nurses working at primary care health centres | Sweden | EHR | Nurses experienced that the EHR service had increased their workload. It sometimes generates duplication of work. |  |
| Coorey et al (62), 2019  MMAT score: 3 | Mixed methods: 1. RCT  2. survey  3. web analytic data  4. interview  5. focus group | Survey: 38 GPs.  Interviews: 17 GPs. | Australia | EHR | GPs mentioned that the use of an EHR saved a lot of time, but also that it was associated with time issues in general practice and time needed to familiarize self, and that there is no additional time to monitor another thing. |  |
| Coylewright et al (82), 2019  MMAT score: 3 | Clinician encrypted information and survey | Encrypted information: 1,209 PCPs. Survey: 109 PCPs. | US | EHR (functionalities) | The majority of PCPs said that they felt visits with an integrated patient decision aid (iPDA) took a similar amount of time, or were more efficient, than usual care. | Adoption was achieved by 657 clinicians for the 8 year period. On average, 82 new clinicians adopted the iPDAs each year (range 56–108). |
| Donohue et al (89), 2015  MMAT score: 3 | Survey | 72 PCPs (mainly family medicine) working in healthcare organizations and communities | US | EHR | Few felt using EHR-generated plans would disrupt clinic workflow or take too much time. A number of PCPs felt that shorter plans would facilitate use. |  |
| Emani et al (73), 2015  MMAT score: 4 | Survey | 853 physicians at two academic medical centers | US | EHR (functionality) | Most physicians believed that generating and providing the after visit summary (AVS) functionality had a negative impact on their workload and their workflow. | Impact of AVS on workload was significantly associated with gender and with the number of hours worked per week. |
| Embi et al (72), 2013  MMAT score: 5 | Focus group | 54 physicians or practitioners, 34 nurses, and 37 administrators from 5 Department of Veterans Affairs facilities | US | EHR (functionality) - computerized provider documentation | Integrating keyboard entry of documentation with workflow was described as time consuming. Clinicians also complained about the time burden of performing computerized provider documentation. Most agreed that the ready availability of historical information made their work more efficient overall. |  |
| Fleming et al (42), 2014  MMAT score: 4 | Interrupted time series design:  1. Billing system data  2. general ledger and payroll system data | 26 primary care practices with physicians specializing in family and internal medicine | US | EHR | Productivity and amount of visits (volume) decreased initially but recovered to/close to pre-implementation levels after 12 months. Visit intensity did not change significantly. |  |
| Gardner et al (51), 2019  MMAT score: 4 | Survey | 1,792 practicing physicians, including PCPs | US | HIT use, including EHR adoption, EHR functionality, electronic prescribing, and HIE | About 70% of physicians with EHRs suffered from HIT-related stress, and the presence of any of the 3 HIT-related stress measures independently predicted burnout symptoms among physicians. |  |
| Ghosh et al (27), 2016  MMAT score: 5 | Focus group | 12 GPs and 13 practice staff from 17 practices | Australia | EHR data management | EHR data management increased practice staff workload. |  |
| Goldberg et al (74), 2012  MMAT score: 5 | Surveys, interviews and observations | 38 clinicians and administrative staff from 6 primary care practices | US | EHR (functionalities) | Concerns about disruption in workflow because of EHR use were reported. |  |
| Green et al (86), 2013  MMAT score: 4 | Surveys | PCPs | US | EHR (functionalities) | The use of PCP pools supported by non-physician professionals and EHRs can be efficient for handling increasing amounts of patients. |  |
| Greenhalgh et al (28), 2010  MMAT score: 2 | Mixed methods:  1. interviews  2. field notes  3. documents  4. stakeholder feedback  5. stakeholder primary care data | Three primary care sites | UK | Shared EHRs | Summary care record use was not associated with shorter consultations. It was characterized by high implementation workload. | High implementation workload should be expected when shared EHRs are introduced on a national scale. |
| Gregory et al (38), 2017  MMAT score: 2 | Mixed methods:  1. survey  2. focus group | 16 PCPs (including physicians, NPs and PAs) from a large medical center | US | asynchronous EHR-related alerts | Subjective, but not objective, alert workload was related to two of the three dimensions of burnout, including physical fatigue and cognitive weariness, when controlling for organizational tenure. | Participants appear to be concerned with objective alert workload, as they desire protected time to respond to alerts, whereas quantitative findings suggest time spent managing alerts was not predictive of burnout. |
| Greiver et al (63), 2011  MMAT score: 5 | Interviews | 12 community-based family physicians | Canada | EHR | An enormous amount of time was initially required for data entry by physicians. Some aspects of the EHR (i.e., prescription refills and consultation letters) made them more efficient after an initial decrease in efficiency. Physicians thought that their administrative personnel were more efficient. |  |
| Halas et al (64), 2015  MMAT score: 5 | Focus groups | 9 physicians (including medical directors), 11 allied health faculty, and 8 family medicine residents from 3 family medicine teaching clinics | Canada | EHR | The EHR typically increased time required for certain processes associated with system performance and clinical work flow. That the EHR saved time was less frequently noted. |  |
| Harle et al (65), 2016  MMAT score: 5 | Several data sources, including:  1. on-site observations  2. interviews  3. (group) discussions  4. demos  5. survey  6. feedback | 6 family medicine practices.  23 providers, 4 IT developers, 4 health system or IT administrators, and 15 practice staff members. | US | EHR (functionality) – Integration of patient-reported outcomes | Time, work flow, and effort constraints represented a barrier that cut across nearly all stakeholders’ feedback on concerns about implementing the system. |  |
| Hauer et al (52), 2018  MMAT score: 4 | Survey | 1,165 practicing physicians from various specialties | US | Information systems, including EHR | Utilization and interactions with electronic health records (EHR) is a primary cause of physician burnout. |  |
| Howard et al (66), 2013  MMAT score: 5 | 1. observations  2. interviews  3. document and photo documentation | Practice members and selected patients in seven community-based primary care practices | US | EHR | EHR use reduced some clinician work, while increasing other work. In general, clinicians reported that tasks involved in chronic disease management and preventive care were more time consuming. Many practice staff members praised their EHR for eliminating some time-consuming tasks. |  |
| Hysong et al (29), 2011  MMAT score: 5 | Focus groups | 44 primary care staff members (PCPs, radiology, information technology (IT) and laboratory services) from two Veterans Affairs facilities | US | EHR (functionality) - test result alerts | Providers reported that their already heavy clinical workloads left very little time for the task of alert management, and contributed to their heavy workload. |  |
| Iljaz et al (30), 2014  MMAT score: 5 | Focus groups | 22 family physicians, 14 nurses and 18 patients | Slovenia | Paperless practices and ICT tools: EHR, e-consultation, e-appointment and e-referrals | The most important identified barriers impeding the use of ICT were: the heavy workload of, and the number of consultations for primary care physicians and nurses, and duplication of work using both paper and EHRs. |  |
| Jabour et al (90), 2020  MMAT score: 2 | Observations | General practitioners from 4 primary healthcare centers | Saudi Arabia | EHR systems | The results showed no significant difference in the amount of time spent by patients in the reception area, in the waiting area, consultation time, and at the pharmacy between the EHR and paper based groups. |  |
| Kersting et al (53). 2019  MMAT score: 3 | Survey | 109 GPs from 61 different general practices | Germany | EHR | Keeping medical records up-to-date had a strong association with high chronic stress among GPs. |  |
| Kim et al (37), 2017  MMAT score: 5 | Interviews | 21 healthcare professionals (10 PCPs, 9 nurse coordinators, and 2 IT/administrative personnel from primary care clinics | US | EHR functions for colorectal cancer screening | Providers strongly felt that being a member of the accountable care organization, using the innovate care delivery model, means more work for all staff members. They acknowledged that they needed to spend extra time and labor for good performance. |  |
| Kochendorfer et al (16), 2010  MMAT score: 2 | Survey | 93 residents and faculty members of the Departments of Family and Community Medicine and Internal Medicine (pre-implementation), 108 residents and faculty members (post-implementation) | US | EHR (functionality) -generated rounding report | After 5 months of use, residents and attending physicians reported a daily time savings of 44 minutes. Rounding report users were more satisfied with the rounding process, spent less time updating other lists or documents, and less time pre-rounding. |  |
| Koopman et al (83), 2011  MMAT score: 4 | 1. observations  2. interviews | 10 UMHS family and general internal medicine physicians with outpatient practices | US | EHR (functionality) – diabetes dashboard / decision support tool | Using a patient-specific diabetes dashboard improves the efficiency of acquiring data needed for high-quality diabetes care. This intervention led to significantly decreased physician time and mouse clicks. |  |
| Krishnan et al (84), 2010  MMAT score: 5 | Interviews | 22 health workers from the Institute of Medical Sciences and Primary Health Centers | India | EHR | The Health Management Information System (HMIS) improves efficiency and saves time for health workers in record keeping and report generation. |  |
| Kroth et al (47), 2018  MMAT score: 3 | mixed methods: 1. focus groups  2. burnout assessment | 40 ambulatory physicians, 1 NP from a large multispecialty group, a federally designated Hispanic serving institution, and a large academic site. | US | health information and communications technologies, including EHRs | Health information and communication technology use has increased the burden of ambulatory practice. Intervention factors associated with physician stress are data entry requirements, inefficiently designed user interfaces, and information overload. Also, productivity went down due to EHR use. |  |
| Kroth et al (54), 2019  MMAT score: 5 | survey | 282 ambulatory primary care and subspecialty clinicians from 3 institutions | US | EHR | 7 EHR design and use factors are associated with high clinician stress and burnout. These 7 plus 2 other design and use factors collectively accounted for a modest amount of the variance in stress and burnout. Other work conditions accounted for considerably more of the variance in stress and burnout. |  |
| Krousel-Wood et al (43), 2018  MMAT score: 4 | survey | 223 academic healthcare providers from 8 hospital and 38 clinics | US | EHR | The percentage of providers with positive perceptions significantly decreased over time, after implementation, for overall productivity and for more time for patients. | Results differed between younger and older providers percentage of providers. |
| Kruse et al (48), 2017  MMAT score: 3 | survey | 333 physicians working at federally operated Indian Health Service facilities | US | EHR (functionalities) | Perceived barriers to EHR use included productivity loss. The EHR increases documentation time and time required to complete a visit. | PCPs more often reported productivity loss as a barrier to EHR adoption, agree that the EHR increases visit time and documentation time, compared to specialist physicians. |
| Lesko et al (44), 2012  MMAT score: 3 | survey | all family medicine residencies in 5 states from two university-based, 14 community-based, and two uniformed services programs | US | EHR | Physician productivity in residency programs decreased for all resident physicians from 2000 to 2010, likely due to a combination of decreased resident duty hours and EHR implementation. |  |
| McAlearney et al (75), 2010  MMAT score: 5 | interviews | 39 clinicians (i.e., physicians, PAs, NPs, and nurses) at each community health center site, all IT staff, laboratory staff, other clinical support staff and administrators. | US | EHR | We found that the implementation of an EHR in community health centers had both positive and negative early impacts on efficiency for those organizations. |  |
| Murphy et al (58), 2012  MMAT score: 3 | Time-motion data | 47 PCPs from a large, tertiary care Veterans Affairs facility | US | EHR alerts | PCPs receive a substantial information load from EHR-based asynchronous alert notification systems and spend a significant proportion of each day processing alerts. | PCPs spent an estimated average time of 49 minutes per day processing their alerts. |
| Murphy et al (31), 2019  MMAT score: 5 | Cognitive walkthroughs | 25 physicians (17 PCPs and 8 specialists) at 6 large healthcare organizations | US | EHR (functionality) | High numbers of clicks are needed to accomplish actions. EHR inbox features and workflows did not always match clinical processes and required more steps than necessary. Some physicians reported increased cognitive load and that it affected their workloads. |  |
| O’Malley et al (87), 2015  MMAT score: 5 | interviews | 22 physicians, 3 NPs, 7 medical assistants, 7 RN/LPN, 9 practice managers, 12 administrative and front desk staff, 3 national experts from 27 primary care practices | US | EHR | According to one practice, EHR use can lead to improvements in productivity and improved efficiency. Furthermore, it can offload work from physicians. |  |
| Or et al (76), 2018  MMAT score: 5 | interviews | 8 physicians and 15 clinical assistants from seven private general practice clinics | Hong Kong | EHR | Workflows were slowed, interrupted, or became more complex because of EHR functions that were desired, missing or not ready. Long response times and the instability of the EHR system disrupted the workflow and created unnecessary burdens and workload. |  |
| Paré et al (67), 2014  MMAT score: 5 | Mixed methods:  1. Delphi study  2. survey | Delphi study panel: 21 GPs, survey: 431 family physicians working in medical  practices | Canada | EHR | Barely reported EHR system adoption barriers include: time to enter data into the EHR system and more time spent with each patient due to EHR system usage. |  |
| Paré et al (91), 2015  MMAT score: 4 | survey | 331 family physicians working at private medical practices | Canada | EHR | Family physicians are unsure as to its positive impact on their clinic's workflow, whereas they indicate no improvements with regard to their efficiency as physicians. | Only a minority of family physicians in the study’s sample use most of the features available in their EHR system. |
| Prazeres et al (68), 2014  MMAT score: 4 | survey | 80 GPs and 67 general practice trainees from healthcare center groups | Portugal | EHR | The majority reported that EHR increases consultation time and that using the EHR means less time devoted to the patient. | Results differed between GPs and general practice trainees. |
| Rahal et al (45), 2019  MMAT score: 5 | interviews | 9 PCPs from primary care clinics and family practices. | Canada | EHR (functionalities) | Participants reported that specific EHR features were time consuming, inefficient and changed productivity. Other participants reported that workflow efficiency and patient efficiency were enhanced by specific EHR features. | Effects of EHR use differed between EHR features. |
| Ramelson et al (32), 2018  MMAT score: 4 | surveys | pre-survey: 20 referring clinicians and 41 primary care staff, post-survey: 17 clinicians and 18 primary care staff. | US | EHR (functionality) – referral manager | The implementation of an enhanced referral manager (EHR) system improved efficiency. Practices reported that additional time was required by the staff to monitor the referral queue and follow up as needed, and it may lead to added workload for staff. |  |
| Robertson et al (55), 2017  MMAT score: 4 | survey | 340 residents and 245 teaching physicians from 19 primary care programs. | US | EHR | A majority of respondents attributed burnout to the EHR. The out-of-work hours  spent with the EHR was significantly associated with reported burnout, and reports on the impact of the EHR on burnout. |  |
| Rosemann et al (69), 2010  MMAT score: 5 | survey | 60 PCPs, 202 internal medicine, 59 surgeons, and 86 psychiatrists working in daily ambulatory care. | Switzerland | HIT, especially EHR use | Physicians (especially PCPs) fear that an EHR change is too time consuming. |  |
| Saleem et al (80), 2011  MMAT score: 5 | 1. controlled simulation experiment  2. surveys  3. video recordings  4. debrief interviews. | 12 PCPs from outpatient clinics. | US | EHR (functionality) – clinical decision support | The EHR functionality positively impacted PCPs’ workflow integration. However, the redesigned prototype showed no difference in terms of perceived workload. |  |
| Sarma et al (70), 2013  MMAT score: 5 | survey | 2,459 family physicians / GPs in 2007 and 4,003 family physicians / GPs in 2010 | Canada | HIT, with or without EHR use (including e-mail, online access, electronic patient appointment, telemedicine) | Use of EHR, HIT and EHR+HIT is found to be associated with fewer patient visits and longer visit length among family physicians relative to NO users in 2007. In 2010, EHR was not statistically significant and EHR + HIT was associated with a slight increased visit length. | Particularly, physicians using EHRs were undertaking about 5 fewer patient visits per week and those using EHR + HIT were undertaking in the range of 9 to 11 fewer patient visits compared to NO users. |
| Shanafelt et al (56), 2016  MMAT score: 4 | survey | 6,375 Physicians including 510 family medicine physicians working in primary care and specialty care | US | electronic environment:  1. EHR (functionalities)  2. CPOE  3. electronic patient portals | EHRs users had lower satisfaction with the amount of time spent on clerical tasks and had higher rates of burnout. | The majority of physicians disagreed with the believe that EHRs improved their efficiency. |
| Shield et al (33), 2010  MMAT score: 3 | Mixed methods:  1. interviews  2. focus groups  3. observations  4. timed tasks measurements | Observations: 13 faculty physicians and 13 residents,  Focus groups: 20 Family Care Center staff | US | EHR (functionalities) | Implementation of an EHR saved time for nurses and improved work efficiencies and workflows. During transition, however, increased staff workload was reported. | Effects of EHR implementation on workload differed during the different stages of EHR implementation. |
| Silva et al (34), 2018  MMAT score: 5 | 1. interviews  2. observations | 62 health professionals from the family health units | Brazil | EHR | Duplicate typing, rework and work overload were reported because of implementation. |  |
| Straus et al (39), 2011  MMAT score: 5 | Interviews | 23 participants (physicians, NPs, administrative staff members, and office managers from specialty clinics. | US | EHR (functionality) - e-referral and e-consultation system | PCPs reported increased workload due to a shift in tasks from specialists and administrative personnel and issues leading to extra time and effort for users of the system. However, also clinical and administrative efficiencies were reported, mainly resulting in time-savings. |  |
| Tierney et al (71), 2016  MMAT score: 4 | 1. time-motion data  2. surveys | 72 635 primary care patients and 15 records officers, 14 nursing officers, 8 clinical officers, 5 data assistants, 3 physicians, 2 administrators, 1 laboratory technician, 1 nutritionist, and 1 pharmacy technician from three Kenyan rural health centers | Kenya | EHR | The time-motion studies showed that patient time in the health centers increased dramatically after EHR implementation.  The number of patient visits per month increased dramatically between the pre- and post-EHR periods. | Effects of EHR implementation on workload differed between health center sites. |
| Tran et al (57), 2019  MMAT score: 5 | 1. survey  2. audit log data | 107 PCPs at 10 university-affiliated primary care clinics. | US | EHR (related tasks) | In general, providers who self-reported burnout were more likely to spend more time in the EHR after hours. |  |
| Yan et al (46), 2012  MMAT score: 5 | 1. survey  2. licensure data | 1,888 PCPs and other specialties | US | EHR | Respondents perceived EHRs’ training and productivity impact as the largest barrier. Further, an increase in the work of documentation was cited. | Wide variation in perceptions of barriers among different physician specialties were found. |
| Zazove et al (36), 2017  MMAT score: 5 | Interviews | 61 clinicians from 2 family medicine clinics | US | EHR functionality – electronic clinical alerts | Time pressures in primary care and clinic workflow variations impeded alert use. Clinicians felt that patient visits were already overloaded, limiting their ability to handle additional alerts. The added workload of alerts contributes to clinician stress. Medical assistants often queue up alerts inappropriately, causing clinician rework. |  |
| Zhang et al (35), 2016  MMAT score: 5 | interviews | 21 PCPs (21 physicians and 2 NPs) from 4 outpatient primary care clinics | US | EHR | The study revealed an increased workload that PCPs face with EHR use. |  |

**Explanation MMAT scores:** 5 = 100% quality criteria met, 4 = 80% quality criteria met, 3 = 60% quality criteria met, 2 = 40% quality criteria met, 1 = 20% quality criteria met

**S table 2** Overview main characteristics included studies – digital communication services

| Study ID | Methods / Design | Participants | Country | Intervention | Outcome measure(s) and key result(s) | Notes |
| --- | --- | --- | --- | --- | --- | --- |
| Adamson et al (174), 2010  MMAT score: 4 | User data from pilot platform | 4,282 patients and 56 clinicians practicing in 4 clinics. | US | e-visit (store and forward) | E-visits made office visits unnecessary in 40% of the cases; in 13% of the cases, the patient was asked to schedule an appointment for a face-to-face encounter. Further, the intervention saved time on simple administrative tasks and clinicians were not overburdened by this intervention. |  |
| Atherton et al (149), 2018  MMAT score: 5 | Mixed methods:  1. observations  2. conversations  3. interviews | 45 staff members, 39 patient and carer participants from general practices | UK | email, e-consultation, and internet video | Unintended consequences such as an increased workload were reported. More specifically, video consultations were found to be time consuming to set up. | Findings suggest that the impact on patterns and volume of workload is complex and reductions in workload cannot be assumed. |
| Atherton et al (150), 2013  MMAT score: 5 | Interviews | 10 GPs, 14 patients, and six experts working in a primary care setting | UK | email | Workload difficulties arose because of email adoption, time is not allowed for ‘extra’ consultations and, in some cases, GPs reported that email was generating more work and additional consultations. |  |
| Banks et al (151), 2018  MMAT score: 5 | Interviews | 23 general practice staff members from 6 general practices | UK | e-consultation | The study found a perceived increase in workload and no improvements in freeing up GP time overall. E-consultations could save clinician time, however, when they were actioned without direct contact between GP and patient. |  |
| Bavafa et al (152), 2019  MMAT score: 4 | Primary care patient encounter data | 368 physicians and 313.655 patients from a large primary healthcare system | US | e-visits (store and forward) | E-visit adoption nearly doubles the number of hours containing work each week. |  |
| Bishop et al (153), 2013  MMAT score: 5 | interviews | 21 medical groups, six providers and non-provider staff | UK | electronic communication (store and forward) | Electronic communication increases the volume of physician work unless office visits are reduced. Providers lamented that the intervention made the workday longer. Some providers also reported related efficiency. |  |
| Carter et al (154), 2018  MMAT score: 4 | Mixed methods:  1. retrospective electronic data  2. case report forms  3. survey  4. interviews | Case reports: 20 GPs Survey: 81 patients  Interviews: 5 GPs and 5 administrators from 6 GP practices | UK | e-consultation | E-consultations had no discernible impact on practice workload. Some interviewees suggested that e-consultations had resulted in an increased workload for the administrative team. | The completeness of cross-sectional data on consultation workload varied between practices. |
| Casey et al (155), 2017  MMAT score: 5 | Mixed methods:  1. interviews  2. scrutiny of documents, websites and demonstrator versions | 3 development / operational staff and 4 end-users (2 GPs and 2 administrators) from 2 GP practices | UK | Online consultation system (store and forward) | The administrative burden because of the online consultation system was substantial. GPs welcomed varied modes of consulting, but the aspiration of improved efficiency was not realized in practice. | The intervention may offer a welcome variation to GPs’ workload. |
| Cowie et al (175), 2018  MMAT score: 3 | Mixed methods:  1. log data  2. surveys  3. interviews  4. focus group | surveys: 291 patients,  interviews: 44 GP staff, focus group: 4 GP staff from 11 GP practices | UK | e-consultation | Results suggest that workload was not decreased through e-consultation use and it has the potential to save time for physicians. |  |
| Daniel et al (156), 2018  MMAT score: 2 | survey | 238 physicians from a large tertiary care academic medical center | Lebanon | Virtual communication (store and forward) | About 70% of the physicians indicated that the intervention provides quicker and more efficient communication between physicians. About 40% of them indicated that it increased their workload. |  |
| Dash et al (176), 2016  MMAT score: 4 | survey | 372 PCPs (including general internists, pediatricians and medical practitioners) | Switzerland | email and text messages | Email exchanges helped physicians save time. Time saving was also the main advantage associated with using text messages. |  |
| Dixon et al (177), 2014  MMAT score: 3 | Survey | 9 physicians, 1 NP and 293 patients from a general hospital ambulatory internal medicine practice site | US | Asynchronous virtual care tool: virtual visits, e-consults, video consults | Asynchronous virtual visits are an effective way to evaluate and manage patients, while providing physicians significant time savings. The clinician time spent for the overall encounter was significantly shorter than for an in-person follow-up visit. |  |
| Donaghy et al (181), 2019  MMAT score: 5 | Interviews | 21 patients, 10 participating GPs, and 3 practice nurses from 6 general practices | UK | Video consultation | Clinicians felt that video consultation use would be time neutral for them (with the possible exception of house visits). |  |
| Edwards et al (182), 2017  MMAT score: 5 | 1. general practice data  2. Website analytics data | 36 GP practices covering 396,828 patients | UK | e-consultation | Any impact of e-consultations on staff workload is likely to be negligible. |  |
| Fagerlund et al (178), 2019  MMAT score: 5 | Interviews | 9 GPs from different GP offices | Norway | e-consults, e-booking, e-prescription, text-based non-clinical enquiries service | Reduced phone load, increased efficiency, released time for medical assessments, a reduction in the number of visits and phone contacts was reported. |  |
| Farr et al (157), 2018  MMAT score: 3 | Mixed methods:  1. interviews  2. survey  3. patient records | 23 GP staff members from 6 GP practices, 485 patient records, 751 patient surveys | UK | e-consultation | Most e-consultations resulted in either follow-on phone or face-to-face appointments, and GPs felt that this duplicated their workload. |  |
| Garrido et al (183), 2014  MMAT score: 3 | Retrospective observation | 3,176 PCPs (physicians, NPs, and PAs) in family practice, internal medicine, pediatrics, and obstetrics/gynecology | US | Secure email | Data suggest that the average impact on physician workloads is manageable. |  |
| Grembowski et al (158), 2012  MMAT score: 5 | Enrollee utilization data | 9,871 Group Health members (patients) from 21 Global Health clinics. | US | Patient portal, including secure messaging | Primary care visits declined, but primary care contacts grew, largely due to the introduction of secure messaging. |  |
| Gulzar et al (159), 2013  MMAT score: 5 | Interviews | 4 Community Health Nurses, 2 Lady Health Visitors, 2 midwives, and 1 field Officer from 4 primary healthcare centers, 3 secondary care centers and 1 medical center | Pakistan | Teleconsultation (store and forward) | Nurses mentioned about their increased workload following the introduction of eHealth and suggested to get protective time for eHealth related activities. |  |
| Hernandez et al (160), 2018  MMAT score: 3 | Survey | 70 providers and staff; 1,260 patients from five family health clinics | US | Secure messaging | The majority of providers agreed that the intervention had increased their workload. Further, a similar proportion of providers that agreed, disagreed that it improved their available time during the duty day. |  |
| Hoonakker et al (161), 2017  MMAT score: 4 | 1. observation  2. interviews  3. survey | survey: 43 clinicians and 15 clinic staff,  observations + interviews: 39 clinicians, 13 staff, patient interviews: 27 patients from 5 clinics | US | Secure messaging | Secure messaging is preferred to phone calls at least some of the time, finding that it reduces workload and makes workflows more efficient. Secure messaging can also add to the workload, especially if there is high message volume. Overall, providers feel that secure messaging shifts work from nurses to providers, increasing their workload. |  |
| Iljaz et al (30), 2014  MMAT score: 5 | Focus groups | 22 family physicians, 14 nurses and 18 patients | Slovenia | Paperless practices and ICT tools: EHR, e-consultation, e-appointment and e-referrals | The most important identified barriers impeding the use of ICT were: the heavy workload of, and the number of consultations for primary care physicians and nurses, and duplication of work using both paper and EHRs. |  |
| Johnson et al (162), 2014  MMAT score: 4 | Interviews | 27 PCPs, including family physicians from Kaiser Permanente and Group Health | US | secure e-mail | The majority of PCPs completed secure e-mail during working hours. The secure e-mail functionality however led to concerns about workload and volume overload. |  |
| Kroth et al (47), 2018  MMAT score: 5 | mixed methods: 1. focus groups  2. burnout assessment | 40 ambulatory physicians, 1 NP from a large multispecialty group, a federally designated Hispanic serving institution, and a large academic site. | US | health information and communications technologies, including EHRs | Electronic messaging was reported as efficient and time-saving by one respondent. |  |
| Lacasta Tintorer et al (179), 2018  MMAT score: 5 | 1. focus groups  2. triangular groups  3. interviews | 29 healthcare professionals (21 physicians and 8 nurses) from 10 primary care centers and 3 secondary care centers | Spain | Online communication tool (store and forward) | The tool is valued for its efficiency and it is useful for improving time management; the participants often identified the lack of time as the main determining factor of using the tool for resolving queries. |  |
| Lee et al (163), 2018  MMAT score: 5 | Interviews | 20 DHS and 20 non-DHS professionals; 29 physicians and 11 advanced practice clinicians from hospitals and primary care sites | US | e-consultation | Most PCPs were frustrated with the administrative burden of e-consultations. Other PCPs believed that the previsit requirements requested by specialty reviewers were a burdensome shift of work to PCPs. |  |
| Lieu et al (164), 2018  MMAT score: 5 | Interviews | 24 physicians (9 chiefs, 15 PCPs) from 8 medical centers | US | electronic communication (store and forward) | All participants described electronic messaging as having led to increased work outside normal work hours. |  |
| Moffatt et al (165), 2011  MMAT score: 5 | Interviews | 5 medical practitioners, 2 medical practitioners, 2 academics, 1 internet specialist, 1 trainer | Australia | Telemedicine: email, direct file transfers, video-conferencing, and webinars | The extra time required for a telemedicine consultation has particular implications for the workload of rural doctors. |  |
| Nazi et al (166), 2013  MMAT score: 5 | Mixed methods: 1. Interviews  2. document and artifacts analysis | 30 VA healthcare professionals (10 healthcare providers, 10 nurses, and 10 pharmacists) | US | Personal health record and secure messaging | Healthcare professional expressed concerns about workload with increased use of secure messaging, as it is now still manageable. Healthcare professionals further report that secure messaging increases efficiency by avoiding telephone tag. |  |
| North et al (184), 2014  MMAT score: 4 | retrospective cohort study using primary care practice data | 121 staff physicians, 59 midlevel providers, 2,357 primary care patients from 3 primary care practices | US | patient portal - secure messaging and e-visits (store and forward) | Secure messaging and e-visits through a patient portal may not result in a change of adult primary care face-to-face visits. |  |
| Ozkaynak et al (167), 2014  MMAT score: 5 | interviews | 29 PCPs, PNs, medical assistants, registered nurses, pharmacists, NP, and social worker) of 8 primary care teams from 2 Veterans Health Administration facilities | US | Secure messaging | As secure messaging was not integrated into the EHR, this led to additional work. Further, eight workflow issues were reported that emerged with the implementation of secure messaging, including additional workload. Despite the significant impact on workload, there was no workload credit for secure messaging. |  |
| Padman et al (180), 2010  MMAT score: 1 | Mixed methods: 1. Usage analysis  2. survey  3. interviews | Survey: 11 physicians,  Interviews: 3 office staff and 3 nurses | US | e-visit (store and forward) | Office staff perceived that the number of phone calls had reduced since the start of the e-visit pilot. |  |
| Palen et al (185), 2012  MMAT score: 4 | survey | 205 PCPs (internal and family medicine physicians) and 267 patients from a large group model practice | US | Virtual consultations via secure messaging | Only 2.4% of respondents indicated that the consultation process disrupted their workflow, with no difference between VCs and TCs. |  |
| Popeski et al (168), 2015  MMAT score: 4 | Mixed methods: 1. survey  2. focus groups  3. interviews | Diabetes care providers (9 physicians and 7 allied health professionals from a centralized diabetes care and education center | Canada | e-mail (exchange): e-consult, e-communication | Allied health professionals reported that responding to an e-mail rather than a voicemail would take less time, whereas more physicians reported that time to responding to an e-mail would be similar to delivering a phone call or that it would take more time. Further, the use of clinical e-mail can improve the efficiency of information exchange. | Significant differences were found between physicians and allied health professionals concerning the use of e-mail and the amount of time spent using e-mail to communicate to patients. |
| Sampson et al (169), 2016  MMAT score: 5 | Interviews | 10 GPs and 12 hospital consultants working in primary and secondary care | UK | Email communication | Asynchronous e-communication allowed for a more considered response, and some flexibility in managing workload. When ‘quick answers’ were not obtained, this led to increased GP frustration and workload. |  |
| Sarma et al (70), 2013  MMAT score: 5 | survey | 2,459 family physicians / GPs in 2007 and 4,003 family physicians / GPs in 2010 | Canada | HIT, with or without EHR use | Use of EHR, HIT and EHR+HIT is found to be associated with fewer patient visits and longer visit length among family physicians relative to NO users in 2007. In 2010, EHR was not statistically significant and EHR + HIT was associated with a slight increased visit length. | Particularly, physicians using EHRs were undertaking about 5 fewer patient visits per week and those using EHR + HIT were undertaking in the range of 9 to 11 fewer patient visits compared to NO users. |
| Sims et al (170), 2016  MMAT score: 5 | survey | 63 physicians, 13 residents, 1 fellow, and 8 NP from the division of Pediatric Neurology and Departments of various specialties, including Family Medicine | US | Teleconsultation (real time) | Perceived barriers to adoption of the teleconsultation intervention included interruptions in workflow. A small majority of clinicians surveyed indicated that it could negatively affect their productivity. |  |
| Tuot et al (171), 2018  MMAT score: 4 | 1. survey  2. focus groups | Over 1,600 PCPs (family physicians, internal medicine physicians, NPs, PAs, general pediatricians) from 4 health systems | US | e-consultation | Implementation barriers common to all four sites included increased and/or different PCP and specialist workload. |  |
| Vydra et al (172), 2015  MMAT score: 2 | 1. personal health record utilization data  2. surveys  3. focus group | Survey: 54 PCPs,  Focus group: 4 PCPs from primary care clinics | US | Personal health record, including secure messaging, request renewal of prescription medications and e-appointments | While PCPs expended significant time communicating with patients using the portal,  they generally overestimated the time spent per week on the system. PCPs raised concerns about the extra time commitment of attending to electronic communication. | Physicians who had been in practice longer estimated a higher average time spent on the system when compared to newer physicians. |
| Zallman et al (173), 2020  MMAT score: 5 | 1. electronic consultation database  2. focus group  3. survey | 41 PCPs (including family medicine) from a safety-net community teaching healthcare system. | US | e-consultation | E-consultation use increased PCP workload, by leading to extra work and required time to write messages. |  |

**Explanation MMAT scores:** 5 = 100% quality criteria met, 4 = 80% quality criteria met, 3 = 60% quality criteria met, 2 = 40% quality criteria met, 1 = 20% quality criteria met

**S table 3** Overview main characteristics included studies – digital decision support services

| Study ID | Methods / Design | Participants | Country | Intervention | Outcome measure(s) and key result(s) | Notes |
| --- | --- | --- | --- | --- | --- | --- |
| Abidi et al (92), 2013  MMAT score: 5 | 1. think aloud observations  2. surveys | 10 family physicians from licensed family physicians' offices | Canada | clinical decision support tool | Our study suggested that although the participant family physicians are potential users of the clinical decision support software like COMET, the most common usability problems encountered are related to time and effort for data entry. |  |
| Barrett et al (105), 2019  MMAT score: 4 | Mixed methods: 1. Survey  2. focus groups | 3 PCPs from 3 different primary care clinics | US | mobile application for clinical pathways | Compared with current clinical tools, participants felt the pathways decreased time spent making clinical decisions. |  |
| Campbell et al (93), 2014  MMAT score: 5 | Randomized trial data | 16,211 patients from 42 GP practices | UK | Computer-supported telephone triage | Compared with usual care, introduction of nurse triage was associated with an increase in the number of primary care contacts. Nurse triage was also associated with an overall increase in total primary care workload. However, it too was associated with a reduction in GP contacts. | There was a redistribution of GP workload from face-to-face to telephone consultations and a redistribution of workload from GPs to nurses. |
| Curry et al (94), 2011  MMAT score: 4 | Mixed methods: 1. decision support software data  2. self-rating analog scale  3. interviews | 16 GPs, family medicine physicians, and surgeons from a family medicine clinic | Canada | Decision support | The time required to interact with decision support was also perceived by physicians to be too long. Initially, 40% reported decision support disruptive in their work flow, which dropped to 16% as physicians gained experience with the software. |  |
| Dikomitis et al (95), 2015  MMAT score: 5 | interviews | 23 GPs | UK | Electronic risk assessment tools – decision support | The vast majority of interviewees asserted that they experienced information overload, which could lead to ‘prompt fatigue’. |  |
| Feldstein et al (96), 2013  MMAT score: 5 | interviews | 52 staff (17 physicians, 4 allied health providers (NPs and PAs), 11 medical assistants, and 20 other managerial staff | US | patient panel-support tool | Tool use during patient visits can increase daily workload and time spent with each patient during office visits. Use for outreach purposes can create clinician access issues and increase ongoing workload. However, many also noted that the tool increased efficiency. |  |
| Hoonakker et al (97), 2012  MMAT score: 2 | 1. time study data  2. observations  3. interviews  4. survey | Time study, interview, and observation data: 8 primary care clinics.  Survey: 130 PCPs | US | Computer decision support tool | A main barrier found was that the tool is relatively time consuming and thus it is difficult to fit in a physician’s workflow. |  |
| Lam Shin et al (98), 2020  MMAT score: 3 | prospective cohort study:  1. system logs 2. chart reviews | 490 patients and 121 PCPs from 3 primary care sites | US | computerized clinical decision support systems | Reported usage barriers included time. Further, a third of users indicated that the decision support process was too time consuming, while slightly more respondents disagreed that it was too time consuming. |  |
| Lemke et al (99), 2020  MMAT score: 5 | interview | 24 PCPs from 14 primary care practice sites | US | a screening tool utilizing the EHR and clinical decision support | A number of challenges were reported during early implementation: a lack of time to use the tool and clinical decision support information in a busy clinical practice and impact on workflow. |  |
| Litvin et al (100), 2016  MMAT score: 2 | interviews | 12 practices representing 25 physicians, 15 midlevel providers | US | clinical decision support | Clinical decision support tools required “extra clicks”. Some providers also felt that use of the tools required additional steps outside the existing workflow. |  |
| Mensah et al (110), 2015  MMAT score: 2 | observations | Start: 108 healthcare providers,  End: 58 healthcare providers (midwives/nurses) from rural primary healthcare facilities | Ghana, Tanzania | electronic clinical decision support system | The most important finding is that the electronic clinical decision support system did not increase the duration of the ANC process at the study sites in Ghana and Tanzania as compared to the non-intervention sites. |  |
| Militello et al (106), 2017  MMAT score: 3 | Survey | 34 PCPs from primary care centers | US | screening and surveillance app | Increases in efficiency and decreases in perceived mental effort and workload were reported when using the Screening and Surveillance App. | Results were compared with using (other) EHRs. |
| Millett et al (101), 2018  MMAT score: 4 | interview | 12 PCPs from academic family practice | Canada | computerized cognitive assessment tools | Every PCP reported insufficient time as a barrier to cognitive assessment. Many PCPs felt that it was difficult to complete cognitive testing and discuss the results during 1 visit.  This meant that additional appointments had to be booked to complete testing. |  |
| Rokstad et al (107), 2013  MMAT score: 4 | interviews | 2,400 patients and 210 GPs | Norway | electronic optional guideline tool | Of the 82 GPs who reported using the tool, 96% found it saved time. |  |
| Schroy et al (108), 2014  MMAT score: 5 | survey | 29 PCPs from a medical center and community health center | US | screening computer-based decision aid | The majority (64%) felt that the use of the tool saved time. Decision aids for screening can improve the efficiency of shared decision making from the provider perspective. |  |
| Shao et al (102), 2015  MMAT score: 5 | 1. interviews  2. focus group | 40 primary healthcare workers from 6 public primary health facilities | Tanzania | electronic decision support (tablets and smartphones) | The majority stated that the devices increased the consultation duration compared to routine practice, because, for example, they have to enter the data in the phone, and then write the same data in the file, or patient’s card/notebook. |  |
| Shibl et al (103), 2013  MMAT score: 5 | interviews | 37 GPs | Australia | Decision Support Systems | Using decision support systems caused more time to be spent in the consultation. Users did not consider workflow influential in their use of decision support systems. |  |
| Williams et al (104), 2020  MMAT score: 2 | Mixed methods:  1. implementation  2. survey  3. interviews | Implementation: 60 PCPs (pediatricians and family-medicine physicians),  1^st^ Survey: 44 PCPs,  Final survey: 39 PCPs, Interviews: 12 PCPs from 2 primary-care (ambulatory) clinics | US | EHR enabled decision-support tool | Provider interviews indicated that useful features of the tool did not increase cognitive workload. However, its use required more time from nurses.  On average, using this tool: 1. enabled to accomplish tasks more quickly, 2. saved time, and 3. increased productivity.  The biggest barrier identified was that it slowed down the workflow and increased clinic visit time for some providers. |  |
| Wu et al (109), 2013  MMAT score: 4 | 1. interviews  2. surveys | 14 PCPs and 1,184 patients from 3 community-based primary care practices | US | family health history collection, risk stratification, and clinical decision support tool | The intervention can be implemented without disruption to workflow. It was able to provide a valuable and time saving resource. |  |

**Explanation MMAT scores:** 5 = 100% quality criteria met, 4 = 80% quality criteria met, 3 = 60% quality criteria met, 2 = 40% quality criteria met, 1 = 20% quality criteria met

**S table 4** Overview main characteristics included studies – telemonitoring services

| Study ID | Methods / Design | Participants | Country | Intervention | Outcome measure(s) and key result(s) | Notes |
| --- | --- | --- | --- | --- | --- | --- |
| Cund et al (124), 2015  MMAT score: 3 | 1. survey  2. qualitative comments | 37 patients and 33 community nurses from 3 locations | UK | Simple TeleHealth “Florence” (software-based short message service texting system to monitor conditions) | The results show that staff did notice a reduction in consultation time, highlighting the potential of the system to save time. The majority of patients find that the service reduced the number of contacts they had with their doctor. |  |
| Davidson et al (125), 2019  MMAT score: 3 | Glucose meter data | 47 patients, 1 clinical NP and a staff person from a safety net family clinic | US | Remote glucose monitoring containing computerized algorithms | Remote glucose monitoring utilizing computerized insulin dose adjustment algorithms saved time for both providers and patients. |  |
| Fairbrother et al (111), 2014  MMAT score: 5 | interviews | 18 patients, 5 professionals (GPs and nurses) from GP practices | UK | Telemonitoring services | Professionals expressed the view that telemonitoring added to workload. Some thought that telemonitoring had the potential to increase rather than diminish the number of home visits (given follow-up requirements in response to telemonitoring data). |  |
| Goodrich et al (112), 2011  MMAT score: 4 | Mixed method: 1. Interview  2. survey,  3. online usage logs | 6 pairs of 6 physicians and 7 medical assistants and 6-8 adult patients from 2 family medicine clinics in a large healthcare system | US | internet-mediated walking program | The intervention is not necessarily considered a time saver. Workflow concerns and, in particular, concerns about increased workload were frequently mentioned by providers. |  |
| Grant et al (113), 2019  MMAT score: 5 | Interviews | 23 patients, 11 GPs, 4 PNs, and 2 patient caregivers from 15 practices | UK | mHealth application, including self- and telemonitoring | Notably only a few of the healthcare providers stated that the intervention was time consuming. | Paper-based recording, however, integrated better into current workflows but required additional staff input. |
| Hanley et al (114), 2013  MMAT score: 5 | interviews | 25 patients, 11 nurses and 9 doctors from 6 primary care practices | UK | telemonitoring | The intervention can increase workloads. Telephone contact increased although it was also found to be a time consuming way of reaching patients who were frequently not available during working hours. |  |
| Hans et al (115), 2018  MMAT score: 5 | 1. focus groups  2. training sessions  3. issue tracker reports | Patient focus group: 12 patients,  provider focus group: 6 PCPs from a family health team | Canada | Electronic patient-reported outcome mobile application and portal system, with remote monitoring | Interoperability between the app and the EHR was found to increase documentation workload during the patient visit. Providers questioned whether the app would actually improve workflow functions or simply add another task. |  |
| Kahalnik et al (126), 2019  MMAT score: 5 | interviews | 11 PCPs from 9 primary clinic sites | US | web-based, self-report based software program for patient screening and monitoring | Providers reported a seamless integration of the technology into the clinic’s existing workflow, and nearly every provider mentioned time as a significant constraint in reference to various aspects of clinic workflow. |  |
| MacNeill et al (116), 2014  MMAT score: 5 | interview | 13 community matrons, 10 telehealth monitoring nurses, and 9 GPs from 3 primary care sites | UK | Telehealth monitoring | Most GPs saw telehealth as increasing their work burden. GPs felt overburdened and undermined and viewed telehealth as just one more imposition on their time. |  |
| Odeh et al (117), 2014  MMAT score: 4 | Interviews | 7 nurses from GP practices | UK | Telehealth monitoring | The most common feature of the nurses’ experiences with telehealth was that it had heavily consumed their time, it affected their daily work and increased their already high workloads. |  |
| Pichayapinyo et al (118), 2019  MMAT score: 2 | interviews | 6 community health nurses and 35 patients from 6 primary care noncommunicable disease clinics | Thailand | interactive voice response based monitoring | Nurses expressed concerns regarding increased workload. |  |
| Sharma et al (119), 2014  MMAT score: 5 | 1. focus group  2. interviews | 7 community matrons, 10 nurses, 5 community support workers, 1 services manager and 1 telehealth/telecare lead from a Primary Care Trust | UK | Telehealth monitoring | The key theme that emerged was that telehealth service perturbed users’ routines and added to their workload. Unnecessary workload demands came from duplicate processes such as the need to enter the same patient information in two data systems. A community matron indicated that it is very time consuming. |  |
| Taylor et al (120), 2015  MMAT score: 5 | interviews | 84 nursing and other frontline staff and 21 managers and key stakeholders from community nursing teams | UK | Telehealth (remote monitoring) | Some participants reported concerns about the potential impact on workload. Monitoring, for example, created additional tasks for existing staff. Others observed resulting efficiencies. Workload associated with telehealth when compared with usual care was viewed as greater. |  |
| Ure et al (121), 2012  MMAT score: 1 | 1. interviews  2. focus group  3. observations  4. utilization data | 20 patients and 25 clinicians and managers (4 GPs, 4 PNs, 2 hospital-based respiratory nurses, 2 nurse managers, 2 physiotherapy managers, 3 physiotherapists, 2 non-clinical managers, and 6 community nurse managers from primary care practices and GP practices | UK | Telemonitoring service | Clinicians had concerns about an increased workload and there was a highly significant increase in primary care consultations (particularly telephone consultations). |  |
| Verwey et al (122), 2012  MMAT score: 5 | interviews | 16 primary care providers (11 nurses, 3 GPs and 2 physiotherapists) from 2 GP practices | The Netherlands | web-based coaching system (for self-monitoring) | Use of the intervention was characterized as being time-consuming. |  |
| Webb et al (123), 2018  MMAT score: 5 | 1. interviews  2. focus groups | 4 GPs and 10 support staff from 1 GP practice clinic | Australia | health and lifestyle screening app | Administering the app presented significant additional work for support staff. Using the app inherently added additional time to a consultation and GPs felt they had to rush to address all issues raised. Indeed, when the app was removed at the end of the study one GP reflected on how it would have saved time to assess a recent patient. | During the 2-month implementation period, the technology and administration of the app was iterated through 4 quality improvement cycles. This resulted in a 4-minute time reduction. |

**Explanation MMAT scores:** 5 = 100% quality criteria met, 4 = 80% quality criteria met, 3 = 60% quality criteria met, 2 = 40% quality criteria met, 1 = 20% quality criteria met

**S table 5** Overview main characteristics included studies – mobile health (mHealth) services

| Study ID | Methods / Design | Participants | Country | Intervention | Outcome measure(s) and key result(s) | Notes |
| --- | --- | --- | --- | --- | --- | --- |
| Diez-Canseco et al (127), 2018  MMAT score: 4 | Mixed methods:  1. web-based data  2. interview | 22 primary healthcare providers (12 midwives, 8 nurses, and 2 nurse assistants) from five primary healthcare centers | Peru | Mental health screening using a mHealth component with a screening app and SMS reminder function | Time constraints and workload were the main barriers to implementing the screening tool. Implementing a new task, such as the screening tool, would involve doing more work within the same time period. |  |
| Ginsburg et al (128), 2016  MMAT score: 5 | 1. usability metrics  2. observations 3. interviews | 30 healthcare providers, 30 caregivers and 9 health administrators from 6 health centers and 5 community-based health planning and services centers | Ghana | mHealth application for diagnosing and treating | The pilot study showed that the mHealth application was thought to be efficient. Thirty percent of the providers noted, however, that the application would add to their workload as it took more time to complete the application steps than standard practice. | These benefits were seen among HCPs with an average of 2 years of professional training. |
| Grant et al (113), 2019  MMAT score: 5 | Interviews | 23 patients, 11 GPs, 4 PNs, and 2 patient caregivers from 15 practices | UK | mHealth application, including self- and telemonitoring | Notably only a few of the healthcare providers stated that the intervention was time consuming. | Paper-based recording, however, integrated better into current workflows but required additional staff input. |
| Gray et al (129), 2016  MMAT score: 0 | 1. focus groups  2. interviews  3. system data | 6 providers and 8 patients from a single interdisciplinary primary healthcare practice | Canada | electronic patient reported outcome tool, linked to mobile device | Provider participants reported experiencing difficulty incorporating patient data into their workflow in terms of: (1) increased charting time required to input data into the provider’s EHR and (2) being able to view data in manageable chunks. |  |
| Hans et al (115), 2018  MMAT score: 5 | 1. focus groups  2. training sessions  3. issue tracker reports | Patient focus group: 12 patients,  provider focus group: 6 PCPs from a family health team | Canada | Electronic patient-reported outcome mobile application and portal system, with remote monitoring | Interoperability between the app and the EHR was found to increase documentation workload during the patient visit. Providers questioned whether the app would actually improve workflow functions or simply add another task. |  |
| Iwu et al (130), 2020  MMAT score: 5 | interviews | 64 healthcare workers from primary healthcare facilities in four subdistricts | South-Africa | mHealth for vaccine stock-levels reporting | Healthcare workers reported extra workload that comes with the system. Most of the participants felt that the intervention poses as an extra burden for them and they felt that sending of reports is time-consuming. |  |
| Mares et al (131), 2016  MMAT score: 2 | Mixed methods:  1. interviews  2. meeting notes  3. focus groups  4. clinician and patient use data | 28 medical providers, 6 nurses, 19 behavioral health workers from 3 primary care health centers | US | mHealth system (smartphone app) for substance use disorders | Workflow and workload concerns were reported after 1-year adoption of mHealth. Some providers, however, reported that it made less work. |  |
| Medhanyie et al (132), 2015  MMAT score: 3 | survey | 15 health extension workers and 10 midwives from 13 primary healthcare facilities | Ethiopia | mHealth forms | Entering the data was time consuming, as both the existing paper forms at health facility and the electronic forms simultaneously had to be completed. |  |
| Pichayapinyo et al (118), 2019  MMAT score: 2 | interviews | 6 community health nurses and 35 patients from 6 primary care noncommunicable disease clinics | Thailand | interactive voice response based monitoring | Nurses expressed concerns regarding increased workload. |  |
| Rothstein et al (133), 2016  MMAT score: 5 | Mixed methods:  1. interviews  2. focus groups | Interviews: 8 community health nurses, 2 midwifes, 2 district health directors, 2 district health information officers,  Focus groups: 15 community health nurses | Ghana | mobile client data app | The app improved community health nurses productivity. It saved time and allowed for more case entries per day and week. Data entry could, however, be time-consuming. | Participants generally reported that the app was easily integrated into their workflow and data capture. |
| Schoen et al (134), 2017  MMAT score: 5 | 1. interviews  2. focus groups | 57 community health workers from 6 primary care centers | Brazil | mHealth tool | The tool helped saving time with bureaucratic paperwork. Overall, participants believed that the mobile application helped make their work more efficient. | Many participants reported finding the mobile application slow. |
| Schooley et al (135), 2016  MMAT score: 5 | mixed-method: 1. Interview  2. survey | 30 GPs and 12 specialists from a single healthcare organization with multiple inpatient and outpatient facilities. | US | Mobile tablet computer connected to electronic health information resources | Physicians found positive gains from utilizing the tablet device in overall productivity, the process of care, and efficiencies. Further, the vast majority of providers believed that it produced no negative impact on workload. Five providers specifically stated that the portability of the device resulted in significant efficiencies. Few participants reported negative impacts including time spent on order submission, note completion time, and overall workload. | Gender, number of years in practice, practice type (general practitioner vs. specialist), and service type (inpatient / outpatient) were found to have a significant effect on provider productivity. |
| Shao et al (102), 2015  MMAT score: 5 | 1. interviews  2. focus group | 40 primary healthcare workers from 6 public primary health facilities | Tanzania | electronic decision support (tablets and smartphones) | The majority stated that the devices increased the consultation duration compared to routine practice, because, for example, they have to enter the data in the phone, and then write the same data in the file, or patient’s card/notebook. |  |

**Explanation MMAT scores:** 5 = 100% quality criteria met, 4 = 80% quality criteria met, 3 = 60% quality criteria met, 2 = 40% quality criteria met, 1 = 20% quality criteria met

**S table 6** Overview main characteristics included studies – electronic prescribing

| Study ID | Methods / Design | Participants | Country | Intervention | Outcome measure(s) and key result(s) | Notes |
| --- | --- | --- | --- | --- | --- | --- |
| Agarwal et al (137), 2010  MMAT score: 5 | Mixed methods:  1. focus group  2. observations  3. interview | Focus group: 13 MDs, 1 NP, 5 OMs and 7 MAs from 21 practices,  Interviews: 2 OMs and 1 MD from 3 practices,  Observations: 10 MDs, 3 residents, 2 OM, 7 nurses, 1 MA from 2 practices | US | Electronic prescribing | Electronic prescribing is seen as an efficiency enhancing tool (i.e. improved productivity) and as an unwelcome disruption (i.e. increased workload). |  |
| Al Alawi et al (59), 2014  MMAT score: 5 | Focus group | 23 PCPs | UAE | EHR, including electronic prescription | PCPs were happy about the electronic prescription option because it saved time. |  |
| Almutairi et al (138), 2018  MMAT score: 4 | Survey | 306 physicians from 25 primary healthcare centers | Kuwait | Electronic prescribing | Most physicians recognized the importance of electronic prescribing to streamline workflow and increase productivity. |  |
| Bulut et al (139), 2018  MMAT score: 5 | 1. Interviews  2. Survey | Interviews: 30 family physicians,  Survey: 1,534 family physicians | Turkey | Electronic prescriptions | The most frequently indicated advantages of the electronic prescriptions were speeding up the prescription process and saving time (36.6% of the respondents). These prescriptions can be generated faster than manual ones. | Although some problems were reported in the first months of the application, family physicians were found to be satisfied with its positive effects on their work and processes. |
| Crosson et al (146), 2011  MMAT score: 4 | Mixed methods:  1. interviews  2. observations | 5 ambulatory primary care practices | US | Electronic prescribing | Successful adoption required substantial investments of planning time and the ongoing transformation of work processes. Many prescribers developed parallel systems for collecting/maintaining medication history data, limiting potential work efficiencies. |  |
| Devine et al (147), 2010  MMAT score: 5 | Time-motion data | 3 primary care sites in a community based, multispecialty health system | US | Electronic prescribing | Electronic prescribing takes longer than handwriting and electronic prescribing at the point of care takes longer than electronic prescribing in offices/workstations. |  |
| Devine et al (140), 2010  MMAT score: 5 | Focus groups | 17 physicians and 53 staff | US | Electronic prescribing | Efficiencies were gained by electronic prescribing by using fewer paper charts. System use was time-neutral or time-saving. Transition required time resulted in workload shift to staff. |  |
| Goldman et al (141), 2010  MMAT score: 2 | Mixed methods:  1. focus groups  2. surveys | Focus groups: 276 persons (clinicians and office staff, survey: 157 clinicians | US | Electronic prescribing | Overall the electronic prescribing functionality saves time in processing refills and related workflow changes reduce the burden of calls to the physician office. |  |
| Jariwala et al (148), 2013  MMAT score: 3 | survey | 443 PCPs (family medicine, general medicine, and internal medicine) | US | Electronic prescribing | Electronic prescribing was associated with workflow problems, time consumption, slow processing, productivity loss, among others. |  |
| Kivekäs et al (142), 2016  MMAT score: 3 | survey | 69 GPs from 2 different primary healthcare organizations | Finland | Electronic prescribing | Electronic prescribing saved time and clarified GPs work. The time required to integrate it into doctor workflow was considered to be a barrier to using the technology. |  |
| Lapane et al (143), 2011  MMAT score: 3 | Mixed methods:  1. focus groups  2. survey | Focus groups: 276 clinicians and office staff  Survey: 157 clinicians | US | Electronic prescribing | Electronic prescribing was reported as being very efficient and timesaving. Improvements in workflow were also noted. Several aspects of the electronic prescribing process were however also considered time consuming and inefficient. | Physicians were more likely to rank electronic prescribing as very efficient relative to non-clinicians. |
| Lichtner et al (144), 2013  MMAT score: 5 | Mixed methods:  1. interviews  2. observations | 4 GP practices | UK | Electronic prescriptions | Use of electronic prescriptions reduced turnaround time and conditioned changes in the workflow, with time-savings found mainly in relation to administrative tasks. But its use also created additional tasks. |  |
| Thomas et al (145), 2012  MMAT score: 5 | survey | 246 healthcare practitioners | US | Electronic prescribing | Most prescribers expected from the electronic prescribing system to improve workflow and efficiency of practice. Most electronic prescribers further expected it will lead to fewer calls from pharmacists. |  |

**Explanation MMAT scores:** 5 = 100% quality criteria met, 4 = 80% quality criteria met, 3 = 60% quality criteria met, 2 = 40% quality criteria met, 1 = 20% quality criteria met

**S table 7** Overview main characteristics included studies – general health information technology services, including telehealth

| Study ID | Methods / Design | Participants | Country | Intervention | Outcome measure(s) and key result(s) | Notes |
| --- | --- | --- | --- | --- | --- | --- |
| Bardsley et al (191), 2013  MMAT score: 4 | Cluster-randomized trial data | 1,219 intervention and 1,098 control patients with diabetes from 179 GP practices | UK | Home-based telehealth | No statistically significant differences were detected in the numbers of GP or PN contacts between intervention and control groups during the trial, or in the numbers of clinical readings recorded on the GP practice systems. |  |
| Dang et al (186), 2019  MMAT score: 3 | Mixed methods: 1. National decision support system data  2. survey  3. interviews | National data: 39,387 Veterans,  Survey: 41 program directors,  Interviews: 18 HBPC program Directors in a home based primary care system. | US | Home Telehealth | Most HBPC staff recognized advantages of using Home TeleHealth, including increased staff efficiency. |  |
| Davidson et al (189), 2013  MMAT score: 5 | Interviews | 20 family physicians, PNs and specialist respiratory physical therapists from general practices | UK | telehealth | Although clinicians were largely positive about integrating telehealth care data into the EHR, they were concerned about the potential increased workload, particularly in respect to error due to data overload. |  |
| Donald et al (187), 2016  MMAT score: 2 | Mixed methods: 1. interviews  2. survey  3. website analytics | Interviews: 10 PCPs, 3 nephrologists, 2 pharmacists and 3 NPs,  Survey: 159 participants,  website analytics: 10,710 visits | Canada | a digital chronic kidney disease clinical pathway website | Having an interactive point-of-care tool accessible by other team members could improve work efficiency. The ability to access the tool by members of the primary care multi-disciplinary team was identified as a positive feature as it assisted in clinic workflow processes. |  |
| Gardner et al (51), 2019  MMAT score: 4 | Survey | 1,792 practicing physicians, including PCPs | US | HIT use, including EHR adoption, EHR functionality, electronic prescribing, and HIE | About 70% of physicians with EHRs suffered from HIT-related stress, and the presence of any of the 3 HIT-related stress measures independently predicted burnout symptoms among physicians. |  |
| Harshbarger et al (190), 2019  MMAT score: 5 | multiple case-study:  1. process evaluation data  2. tool use data  3. interviews | 9 clinical staff persons and 1 HIV PCP, and 104 patients from 4 primary care clinics. | US | Web-based, tailored video counseling tool | The intervention caused delays and the ability to embed the intervention in clinic workflow without disruption, proved to be complex. | Two clinics reported more struggles with implementation. |
| Ruiz Morilla et al (17), 2017  MMAT score: 5 | Survey | 760 practicing physicians from the Barcelona Medical Association | Spain | Telemedicine | Physicians considered that telemedicine would improve the professional workload. |  |
| Sarma et al (70), 2013  MMAT score: 5 | survey | 2,459 family physicians / GPs in 2007 and 4,003 family physicians / GPs in 2010 | Canada | HIT, with or without EHR use | Use of EHR, HIT and EHR+HIT is found to be associated with fewer patient visits and longer visit length among family physicians relative to NO users in 2007. In 2010, EHR was not statistically significant and EHR + HIT was associated with a slight increased visit length. | Particularly, physicians using EHRs were undertaking about 5 fewer patient visits per week and those using EHR + HIT were undertaking in the range of 9 to 11 fewer patient visits compared to NO users. |
| De Wilt et al (188), 2020  MMAT score: 5 | interviews | 10 GPs from 10 GP practices | The Netherlands | eHealth services including patient portals, making appointments online, online medication service, e-consultations, online diagnostic testing | Nine out of the ten GPs experienced that the eHealth services they offered made their job more efficient and run more smoothly. Another GP indicated that online diagnostic testing reduced some of the workload. This also reduces the number of patients that need to visit the office, thereby reducing the workload. |  |

**Explanation MMAT scores:** 5 = 100% quality criteria met, 4 = 80% quality criteria met, 3 = 60% quality criteria met, 2 = 40% quality criteria met, 1 = 20% quality criteria met

**S table 8** Overview main characteristics included studies – patient portal / personal health records

| Study ID | Methods / Design | Participants | Country | Intervention | Outcome measure(s) and key result(s) | Notes |
| --- | --- | --- | --- | --- | --- | --- |
| Delbanco et al (192), 2012  MMAT score: 3 | Surveys | 105 PCPs and 13,564 of their patients from primary care practices | US | Patient online access to visit notes | Most PCPs reported little or no impact on daily workload. Several doctors acknowledged fears about additional time burden. Some commented on the extra time needed for writing, editing, or explaining notes to patients. Few PCPs reported requiring longer encounters, taking more time with visits, or addressing more questions outside of visits. Some cited increased efficiency. |  |
| Fitton et al (193), 2014  MMAT score: 2 | Mixed methods:  1. survey data  2. practice data | 2 urban GP practices | UK | Patient record access | Patient record access is likely to save time for patients and practices. If 30% of patients accessed their electronic general practice record online at least twice a year, a 10 000-patient practice is likely to save 4747 appointments and 8020 telephone calls each year – about 11% of appointments. |  |
| Miller et al (194), 2016  MMAT score: 5 | interviews | 6 nurses, 8 physicians/advanced practice providers, 2 other non-medical clinicians, 4 clinic managers / administration from health centers in 4 counties | US | Patient portals | Portals could improve office efficiency and save time, such as decreasing the volume of incoming phone calls for prescription refills and lab result requests. Many concerns were however also reported, including portals’ potential to generate more work, that these could hamper workflows, increase stress, and introduce extra tasks into their days. |  |
| Pagliari et al (195), 2012  MMAT score: 0 | 1. survey  2. interviews | 14 practice managers, 15 clinicians and 13 patients from primary care centers | UK | Online patient record access | The majority of professionals perceived no increase in the volume of patient queries or clinical consultations as a result of record access; indeed some believed that these had decreased. Overall, it could be provided without creating a significant additional burden on the health center and the majority perceived no adverse impact on consultation length or the frequency of consultations. | Representatives from practices that had opted not to proceed with record access perceived workload (e.g. time required to check records; patients seeking clarification). |
| Palen et al (197), 2012  MMAT score: 4 | EHR administrative data for healthcare utilization | 44.321 patient portal-users and 44.321 non-users from a group model, integrated healthcare delivery system | US | Patient online access system, including e-appointments, medication refill requests, and secure messaging | Having online access to medical records and clinicians was associated with increased use of clinical services compared with group members who did not have online access. Patients with online access to their medical records, including secure e-mail communication with clinicians, had a subsequent increase in use of most in-person and telephone clinical services. | In the year following activation, members with such access had increased rates of office visits, telephone encounters, and acute care services compared with a matched cohort of members without online access. |
| Raza Khan et al (198), 2019  MMAT score: 3 | 1. interviews  2. observations | GPs, other staff and patients from 10 GP practices | Australia | Digital health information system for citizens | Extra workload for administration staff was reported because of digital health information system implementation. | The intervention was not well integrated in GP practices nor the community. |
| Shanafelt et al (56), 2016  MMAT score: 4 | survey | 6,375 Physicians including 510 family medicine physicians working in primary care and specialty care | US | electronic environment:  1. EHR (functionalities)  2. CPOE  3. electronic patient portals | Use of a patient portal was not independently associated with either satisfaction with clerical burden or burnout in adjusted analyses. | The majority of physicians disagreed with the believe that a patient portal increased their efficiency. |
| Sorondo et al (196), 2016  MMAT score: 3 | 1. focus groups  2. interviews | 10 care coordinators, 24 healthcare providers (physicians and NPs), and 92 chronic-condition patients | US | Integrated patient self-reported screening tool in a patient portal | Providers reported feelings of decreased productivity. When compared, the workflow after implementation showed: 1. a reduction of time spent by medical assistants during the office visit, 2. no significant changes in the time spent by providers during the office visit, and 3. an increase of time spent by the office staff, care coordinators, and providers prior to the office visit. |  |
| Vydra et al (172), 2015  MMAT score: 2 | 1. personal health record utilization data  2. surveys  3. focus group | Survey: 54 PCPs,  Focus group: 4 PCPs from primary care clinics | US | Personal health record, including secure messaging, request renewal of prescription medications and e-appointments | PCPs expressed optimism regarding PHR use in office workflows | Physicians who had been in practice longer estimated a higher average time spent on the system when compared to newer physicians. |

**Explanation MMAT scores:** 5 = 100% quality criteria met, 4 = 80% quality criteria met, 3 = 60% quality criteria met, 2 = 40% quality criteria met, 1 = 20% quality criteria met

**S table 9** Overview main characteristics included studies – Health information exchange

| Study ID | Methods / Design | Participants | Country | Intervention | Outcome measure(s) and key result(s) | Notes |
| --- | --- | --- | --- | --- | --- | --- |
| Compeau et al (199), 2017  MMAT score: 2 | Mixed methods:  1. interviews  2. survey | Interviews: 2 PCPs, 2 administrative staff, 1 hospital IT manager,  Group interview: 3 staff members,  Survey: 99 physicians, 1 nurse, 1 NP, and 4 administrators | US | HIE system | The results showed information quality, ease of completing tasks and clinical process improvement as key benefits that reduced workload. Challenges related to system reliability, quality of reports, and service quality increased workload. | The impact of the challenges construct on productivity and workload was lower than the impact of benefits. |
| Mac McCullough et al (200), 2014  MMAT score: 5 | interviews | 24 providers, administrators and office staff from 16 practices and clinics | US | HIE | Instances were found in which the HIE system improved workflow. However, instances were also found in which it appeared to hinder workflow. |  |
| Motulsky et al (204), 2019  MMAT score: 2 | Mixed methods:  1. interviews  2. usage data | 24 GPs, 8 nurses, and 9 pharmacists from 4 family health teams and 2 academic health centers. | Canada | HIE | HIE was perceived as reducing delays related to clinical information management. |  |
| Nagykaldi et al (201), 2014  MMAT score: 3 | 1. time-motion data  2. observations | 346 of 6 primary care practices | US | HIE with intelligence | HIE with intelligence adoption was associated with improved workflow efficiency. Average workload of a clinician practice increased however by about 30 minutes per day postimplementation compared with baseline. |  |
| Rudin et al (202), 2011  MMAT score: 5 | interviews | 15 clinicians and 5 HIE staff from hospital and office-based practices | US | HIE | Clinicians were motivated to access the HIE by perceived improvements in time savings, but their motivation was moderated by an extensive list of factors including workflow issues and usability issues. |  |
| Sandberg et al (203), 2012  MMAT score: 5 | interview | 85 healthcare providers and medical facility staff from hospital and group practices | US | HIE for exchange of medical imaging studies | Many subspecialists and their staff report experiencing difficulty and time delays in accessing and using imaging studies on portable media. The sharing and viewing of medical imaging studies on portable media is often inefficient. | Family medicine practitioners did not identify problems associated with viewing medical images. |

**Explanation MMAT scores:** 5 = 100% quality criteria met, 4 = 80% quality criteria met, 3 = 60% quality criteria met, 2 = 40% quality criteria met, 1 = 20% quality criteria met

**S table 10** Overview main characteristics included studies – algorithms or artificial intelligence

| Study ID | Methods / Design | Participants | Country | Intervention | Outcome measure(s) and key result(s) | Notes |
| --- | --- | --- | --- | --- | --- | --- |
| Bessat et al (205), 2019  MMAT score: 5 | 1. interviews  2. focus groups | Interviews: 12 primary healthcare workers,  Focus groups: 9 primary healthcare workers from 10 health facilities. | Burkina Faso | Electronic clinical decision algorithm | Use of the electronic algorithm could increase consultation duration compared to routine practice but reduced it compared to a paper consultation. Dissatisfaction was reported about work duplication when electronic and paper systems remained running in parallel. |  |
| Davidson et al (125), 2019  MMAT score: 3 | Glucose meter data | 47 patients, 1 clinical NP and a staff person from a safety net family clinic | US | Remote glucose monitoring containing computerized algorithms | Remote glucose monitoring utilizing computerized insulin dose adjustment algorithms saved time for both providers and patients. |  |
| Fabrellas et al (206), 2013  MMAT score: 4 | Electronical medical record data | Patients, nurses and GPs from 284 primary care practices | Spain | Nurse algorithm-guided care | A program of algorithms-guided care sometimes led to significantly increased resolution rates in illnesses, sometimes in decreased rates, and sometimes it did not change significantly. The return to consultation of all minor illnesses together was significantly higher during the second year compared to that of the first year. | The program was devoted to a large number of acute minor illnesses that represent an important workload in primary care. |
| Long et al (207), 2016  MMAT score: 0 | 1. observations  2. interviews | 10 patients, 1 physician and 1 nurse from a family practice clinic | US | medication reconciliation web-based app, artificial intelligence | The tool was easy to integrate into existing workflow and time-saving for the nurse. During the pilot, the tool did not save time for the physician. |  |
| Mason et al (208), 2018  MMAT score: 2 | Mixed methods:  1. search utility data  2. meeting observations  3. interviews | 8 GPs and 10 patients from 8 GP practices | UK | search utility computer application using a search algorithm | GPs were concerned about workload implications of assessment and care planning |  |

**Explanation MMAT scores:** 5 = 100% quality criteria met, 4 = 80% quality criteria met, 3 = 60% quality criteria met, 2 = 40% quality criteria met, 1 = 20% quality criteria met

**S table 11** Overview main characteristics included studies – digital mental healthcare

| Study ID | Methods / Design | Participants | Country | Intervention | Outcome measure(s) and key result(s) | Notes |
| --- | --- | --- | --- | --- | --- | --- |
| Diez-Canseco et al (127), 2018  MMAT score: 4 | Mixed methods:  1. web-based data  2. interview | 22 primary healthcare providers (12 midwives, 8 nurses, and 2 nurse assistants) from five primary healthcare centers | Peru | Mental health screening using a mHealth component with a screening app and SMS reminder function | Time constraints and workload were the main barriers to implementing the screening tool. Implementing a new task, such as the screening tool, would involve doing more work within the same time period. |  |
| Fortney et al (211), 2011  MMAT score: 4 | Budget impact analysis | 395 patients from VA Community-based Outpatient Clinics | US | telemedicine-based mental care | Results suggest that telemedicine-based collaborative care does not increase total workload for PCPs or mental health providers. |  |
| Krog et al (209), 2018  MMAT score: 5 | interviews | 9 GPs from 8 GP practices | Denmark | web-based tool for diagnosis and monitoring of patients with depression | Some of the interviewees experienced that using the tool was time-consuming (login process and filling in the tool). |  |
| Montero-Marin et al (212), 2018  MMAT score: 4 | 1. Face-to-face meeting  2. online practice sessions | 290 GPs | Spain | blended web-based mindfulness intervention | There were no significant improvements in the burnout subtypes, in any of the GP groups |  |
| Sogomonjan et al (210), 2019  MMAT score: 2 | Survey | 18 family physicians, 1 psychologist and 1 psychiatrist working in primary care settings | Estonia | internet-delivered cognitive behavioral therapy (iCBT) | iCBT increases workload in such a way as to lead to inattention. The majority of GPs found that iCBT implementation requires additional training and time. One GP emphasized that iCBT saves healthcare professionals’ time. |  |

**Explanation MMAT scores:** 5 = 100% quality criteria met, 4 = 80% quality criteria met, 3 = 60% quality criteria met, 2 = 40% quality criteria met, 1 = 20% quality criteria met

**S table 12** Overview main characteristics included studies – Digital patient questionnaire (screening) tool

| Study ID | Methods / Design | Participants | Country | Intervention | Outcome measure(s) and key result(s) | Notes |
| --- | --- | --- | --- | --- | --- | --- |
| Lowe et al (215), 2010  MMAT score: 1 | interviews | 18 practice managers from general practices | UK | self-service kiosk technology for measurements | There were significant time savings both for nursing and GP staff. | The benefits of health kiosks appear to increase with time, as clinicians and patients become more familiar with the technology. |
| O’Brien et al (213), 2017  MMAT score: 0 | Mixed methods:  1. survey  2. comparative study  3. interviews | Comparative study: 73 patients in the e-form group and 258 patients in de p-form group,  interviews: 15 patients, 5 administrative staff and 4 PCPs in a community-based setting | US | electronic screening forms | Staff members expressed low administrative burden except for an extra step to link appointment information to patient demographics to identify eligible patients. |  |
| Paul et al (214), 2013  MMAT score: 5 | Survey | 51 GPs and 4058 patients from 12 practices. | Australia | waiting-room touchscreen computers for collection of health behavior information | A majority of the GPs indicated that the operation of the survey was not disruptive to practice. |  |
| Sorondo et al (196), 2016  MMAT score: 3 | 1. focus groups  2. interviews | 10 care coordinators, 24 healthcare providers (physicians and NPs), and 92 chronic-condition patients | US | Integrated patient self-reported screening tool in a patient portal | Providers reported feelings of decreased productivity. When compared, the workflow after implementation showed: 1. a reduction of time spent by medical assistants during the office visit, 2. no significant changes in the time spent by providers during the office visit, and 3. an increase of time spent by the office staff, care coordinators, and providers prior to the office visit. |  |

**Explanation MMAT scores:** 5 = 100% quality criteria met, 4 = 80% quality criteria met, 3 = 60% quality criteria met, 2 = 40% quality criteria met, 1 = 20% quality criteria met

**S table 13** Overview main characteristics included studies – Online test ordering

| Study ID | Methods / Design | Participants | Country | Intervention | Outcome measure(s) and key result(s) | Notes |
| --- | --- | --- | --- | --- | --- | --- |
| Bowie et al (216), 2014  MMAT score: 5 | Focus group interviews | 40 staff members from general medical practices | UK | systems-based management of laboratory test ordering | Contacting doctors to clarify the meaning of the message and also ask the patient to telephone back added to workload and inefficiency. |  |
| Whiting et al (217), 2019  MMAT score: 0 | 1. interviews  2. plan, do, study, act strategy  3. laboratory requesting data | GPs, healthcare assistants, nurses from 20 GP practices | UK | one-click electronic test ordering systems | At the beginning of the project workload was adversely affected across the healthcare system. A systematic approach that aligns the use of common blood tests to valid clinical questions has eventually produced significant reductions in workload. |  |
| De Wilt et al (188), 2020  MMAT score: 5 | interviews | 10 GPs from 10 GP practices | The Netherlands | eHealth services including patient portals, making appointments online, online medication service, e-consultations, online diagnostic testing | Nine out of the ten GPs experienced that the eHealth services they offered made their job more efficient and run more smoothly. Another GP indicated that online diagnostic testing reduced some of the workload. This also reduces the number of patients that need to visit the office, thereby reducing the workload. |  |

**Explanation MMAT scores:** 5 = 100% quality criteria met, 4 = 80% quality criteria met, 3 = 60% quality criteria met, 2 = 40% quality criteria met, 1 = 20% quality criteria met

**S table 14** Overview main characteristics included studies – Teledermatology

| Study ID | Methods / Design | Participants | Country | Intervention | Outcome measure(s) and key result(s) | Notes |
| --- | --- | --- | --- | --- | --- | --- |
| Armstrong et al (218), 2012  MMAT score: 5 | interviews | 10 PCPs working in health centers | US | teledermatology | PCPs identified improved workflow because of teledermatology. |  |
| Janda et al (220), 2019  MMAT score: 3 | survey | 59 healthcare practitioners (GPs (n=17), dermatologists (n=22), dermatology registrars (n=18), a dermatology research fellow (n=1) and a plastic surgeon (n=1)) | Australia | teledermatoscopy | The healthcare practitioners’ most common concerns were time constraints and the ability to handle the extra demand for teledermatology services. |  |
| McFarland et al (219), 2013  MMAT score: 4 | Survey | 21 PCPs and 34 imaging technicians from rural outpatient clinics and parent facilities | US | teledermatology | Implementation challenges reported by providers include an increase in workload. The teledermatology program required extra work for PCPs and imaging technicians. However, 62% of PCPs agreed that teledermatology may actually save time. | Less than half (43%) of providers reported having sufficient amount of dedicated time for the teledermatology process. |

**Explanation MMAT scores:** 5 = 100% quality criteria met, 4 = 80% quality criteria met, 3 = 60% quality criteria met, 2 = 40% quality criteria met, 1 = 20% quality criteria met

**S table 15** Overview main characteristics included studies – Self-management tools

| Study ID | Methods / Design | Participants | Country | Intervention | Outcome measure(s) and key result(s) | Notes |
| --- | --- | --- | --- | --- | --- | --- |
| Gray et al (129), 2016  MMAT score: 0 | 1. focus groups  2. interviews  3. system data | 6 providers and 8 patients from a single interdisciplinary primary healthcare practice | Canada | electronic patient reported outcome tool, linked to mobile device | Provider participants reported experiencing difficulty incorporating patient data into their workflow in terms of: (1) increased charting time required to input data into the provider’s EHR and (2) being able to view data in manageable chunks. |  |
| Poppe et al (136), 2018  MMAT score: 3 | 1. survey  2. interviews | 15 GPs and 232 patients from 19 GP practices | Belgium | Online patient self-regulation and support program | GPs highlighted the difficulty to integrate additional tasks into their daily workflow due to an overload of medical and administrative tasks. Furthermore, it was mentioned that the intervention sometimes required an additional consultation to motivate patients. |  |

**Explanation MMAT scores:** 5 = 100% quality criteria met, 4 = 80% quality criteria met, 3 = 60% quality criteria met, 2 = 40% quality criteria met, 1 = 20% quality criteria met

**S table 16** Overview main characteristics included studies – Other eHealth services

| Study ID | Methods / Design | Participants | Country | Intervention | Outcome measure(s) and key result(s) | Notes |
| --- | --- | --- | --- | --- | --- | --- |
| Albrecht et al (222), 2017  MMAT score: 4 | Surveys | 50 GPs and 170 patients | Germany | Smart devices | One fifth of the GPs were concerned about the possibility of having to invest too much time to become sufficiently familiar with smart devices. |  |
| Bouskill et al (224), 2018  MMAT score: 5 | 1. interviews  2. observations | 23 staff members from 6 ‘safety-net’ clinics | US | Telemedical screening | The implementation of a novel telemedical platform for diabetic retinopathy screening contributes to workarounds that account for additional tasks and patient volume. The presence of additional screening tools places significant burdens on existing care resources. |  |
| Butler et al (221), 2014  MMAT score: 3 | Survey | 353 primary care staff, including providers, nurses, and support staff | US | Virtual quality improvement collaborative | A number of barriers for participating fully in Virtual Collaborative activities, including issues related to staffing constraints, insufficient and/or unprotected time, and competing workload pressures were reported. | Respondents who were fully engaged in the Virtual Collaborative activities found it less disruptive, even though this group presumably devoted more time to the intervention activities. |
| Chiasson et al (230), 2020  MMAT score: 2 | Survey | 51 residents, 45 faculty, 6 NPs, 4 fellows and 5 other roles from 22 family medicine residencies | US | Online education course | The course had no impact on burnout for either group. |  |
| Guldberg et al (223), 2010  MMAT score: 5 | Interviews | 11 GPs from 2 partnership practices 2 solo GPs | Denmark | Electronic feedback system | The electronic feedback system did not communicate with the in-house IT system which resulted in time consuming clicking around on the screen. Use of the system was limited as additional time was required getting to know the new system. |  |
| Ho et al (229), 2013  MMAT score: 2 | 1. surveys  2. logs  3. focus group  4. interviews | 105 GPs and 12 pharmacists | Canada | Technology-Enabled Academic Detailing | Technology-Enabled Academic Detailing is more time efficient compared to Non-Technology-Enabled Academic Detailing for both academic detailers and physicians. |  |
| Irfan et al (227), 2018  MMAT score: 3 | Surveys | 92 residents, 40 family physicians | Saudi Arabia | Social media | Study participants appeared to consider social media as improving job performance and productivity. Physicians found it an effective use of time. | Time constraint was reported as a barrier. |
| Nemeth et al (225), 2012  MMAT score: 2 | 1. observations  2. interviews | 5 family practices, 2 multispecialty, 1 internal medicine with 2 to 25 clinicians per practice. | US | Electronic standing orders | One barrier related to the implementation of electronic standing orders is related to time management concerns of some staff regarding new responsibilities. A facilitator was that staff interaction frees provider to address other health priorities. |  |
| Savoy et al (226), 2018  MMAT score: 5 | within-group experiment data | 30 PCPs working in primary care clinics | US | computer-based prototype consultation order templates | The prototype required significantly fewer mouse clicks than the control. Although overall task time did not differ significantly, the prototype significantly quickened identification of the appropriate specialty clinic. The prototype was significantly associated with a lower perceived workload. |  |
| Schoenhaus et al (228), 2016  MMAT score: 2 | 1. observations  2. survey | 130 PCPs from 14 primary care sites | US | electronic medication refill system | Physicians and clinical staff have estimated that the service provides between 20 and 30 minutes of time savings per day. |  |
| Shanafelt et al (56), 2016  MMAT score: 4 | survey | 6,375 Physicians including 510 family medicine physicians working in primary care and specialty care | US | electronic environment:  1. EHR (functionalities)  2. CPOE  3. electronic patient portals | CPOE users had lower satisfaction with the amount of time spent on clerical tasks and had higher rates of burnout. |  |

**Explanation MMAT scores:** 5 = 100% quality criteria met, 4 = 80% quality criteria met, 3 = 60% quality criteria met, 2 = 40% quality criteria met, 1 = 20% quality criteria met
